# Supplementary material for: Anthropometric analysis of orbital and nasal parameters for sexual dimorphism: New anatomical evidences in the field of personal identification through a retrospective observational study
Source: PLoS One. 2023 May 3;18(5):e0284219. doi: 10.1371/journal.pone.0284219 (PMC10155994; doi:10.1371/journal.pone.0284219)
Supplement: Attachment — Submitted filename: Comments-PONE-D-22-09865.pdf [file pone.0284219.s006.pdf]

PLOS ONE

Anthropometric analysis of orbital and nasal parameters for sexual dimorphism: new anatomical evidences in the field of personal identification

--Manuscript Draft--

**Manuscript Number:**	PONE-D-22-09865R2
**Article Type:**	Research Article
**Full Title:**	Anthropometric analysis of orbital and nasal parameters for sexual dimorphism: new anatomical evidences in the field of personal identification
**Short Title:**	New anatomical evidences for forensic personal identification
**Corresponding Author:**	Francesco De Micco, M.D., Ph.D.<br>Campus Bio-Medico University: Universita Campus Bio-Medico di Roma<br>Roma, Lazio ITALY
**Keywords:**	Orbital index; nasal index; anthropometric parameters; sexual dimorphism; anthropometric study; personal identification.
**Abstract:**	Orbital and nasal parameters among modern humans show considerable variation, which affects facial shape, and these characteristics vary according to race, region, and period in evolution. The aim of the study was to ascertain whether there are sex differences in the orbital and/or nasal indexes (OI and NI) and/or the single measurements used to calculate these in a Kosovar population. The following parameters were taken into consideration: orbital height (OH), orbital width (OW), nasal height (NH), and nasal width (NW). The ratios between orbital index/nasal index (RONI) were calculated. All measurements were obtained from a population sample comprising 408 individuals. The sample was randomly divided into two subsamples, one constituting approximately 2/3 (n=251) of the initial sample, and the other containing approximately 1/3 of the initial sample (n=157). This division was important for the steps of training (2/3 of sample) and testing (validation) (1/3 of sample) variables of interest for the prediction of sex. Therefore, 251 individuals were evaluated, 110 females and 141 males, with a mean age of 37.41 (±20.56) and 33.87 (±19.58), respectively. The data were collected from head CT scans using digital calipers and statistically analyzed (Kolmogorov-Smirnov normality test, Levene's test, Student's t-test and logistic regression). After prediction analysis, the cutoff points were selected: 36.76 for NW measurements, and 50.00 for NH measurements. For two variables, measurements below these cutoff values indicated female sex and measurements above indicated male sex. The accuracy in sex prediction was 52.86% (CI95% = 45.05% - 60.67%) for NW and 64.96% for NH (CI95% = 57.50% – 72.42%). The difference between male and female indexes was statistically significant (P < 0.05). The anthropometric study revealed that only NW and NH are configured as predictors of sexual dimorphism. It could be useful to increase the number of samples to test the discriminant function in other population groups.
**Order of Authors:**	Roberto Scendoni
	Jeta Kelmendi
	Isabella Lima Arrais Ribeiro
	Mariano Cingolani
	Francesco De Micco
	Roberto Cameriere
**Response to Reviewers:**	Dear Editor in Chief<br><br>thank you very much for your kind mail and the precious reviews included in your response.<br>We are glad to inform You that all the criticisms raised by Reviewers have been accepted.<br>The manuscript has been largely revised and improved according to these criticisms.<br>In this regard, we really thank the Reviewers for the relevant improvement to the article

Powered by Editorial Manager® and ProduXion Manager® from Aries Systems Corporation

This is our reply point-by-point.

Reviewer #1

a)Reply to issue no. 1: “Dear author the article you have cited here is speaking of only orbital index and that too not about evoution. please revise and be specific while citing articles”

Thank you for your remark. We have added the following references:

-Oladipo GS, Olabiyi AO, Oremosu AA, NoronhaCC. Nasal indices among major ethnic groups in Southern Nigeria. SRE. 2007; 2(1): 20-22.
-Ravichandran S, Yuvraj Babu K, Mohanraj KG. Correlation of facial and nasal index in gender determination. Drug Invention Today. 2018; 10(12).

b)Reply to issue no. 2: “What were the inclusion and exclusion criteria of the study?”

In the “Materials and Methods” section, we have added the following explanation: “Inclusion criteria were Kosovar males and females over the age of 18, who underwent CT scan to investigate neurological symptoms. Exclusion criteria included growth diseases, endocrine disorders or osteodystrophy, previous fractures of the of the skull and the facial massif. Finally, all blurred TC images were disregarded”

c)Reply to issue no. 3: “How was this sample size calculated?”

In the “Materials and Methods” section, we have added the following explanation: “The sample of the present study was calculated based on the effects observed in the study by Kotian et al. From this study, the mean effect size of difference between groups of 0.20 (Cohen's d) was observed. Adopting a 5% type I error (95% study confidence level), a 20% type II error (80% study power), the effect size observed in the mentioned study and, considering the comparison tests of the groups of the bilateral type (two-sided), it was calculated that at least 394 sample units were necessary for the accomplishment of the study. As they are available, we conducted the study with 408 sample units. The calculations were performed using the G*Power statistical software”

d)Reply to issue no. 4: “Dear author, is this line so important for the result section.”

Thanks for the suggestion. We have deleted the previous sentence and explained our thinking better: “It was not possible to fit a multiple model, with more than one variable being a predictor together with another, among the variables evaluated. For this reason, the univariate analysis is shown in Table 2, where it is observed that, in isolation, the variables NW and NH were configured as predictors of sex”

e)Reply to issue no. 5: “This is not a correct statement over here, the increase in the number of samples depends upon the sample size calculation and in this study the authors have enough sample size. however, the basis of sample taken has not been shown so the methodology clearly needs how the sample size was obtained and then the revision of the line 263 has to be thought of”

The following sentence has been deleted “to increase the number of samples”. The answer to criticism No. 3 shows how the sample was calculated.

f)Reply to issues nos. 6, 7, 8, 9 and 10.

The references have been corrected

***

Reviewer #2

	a)Reply to issue no. 1: “Can you make clear why you choose female sex as a reference group?”<br><br>We have added the following sentence to better explain the reason for the choice: “The female gender was chosen as the reference category because the measures of interest were lower for females and leaving this category as a reference makes the interpretation of the odds ratio findings more understandable”<br><br>b)Reply to issues nos. 2 and 3: “The authors did not establish a correlation between sex difference and mean age why?” and “The authors did not establish a correlation between sex difference and mean age why?”<br><br>Thanks to the reviewer for the remark. The comparison was made and reported in the 'Results' section. Below is the sentence: “251 individuals were evaluated, 110 females and 141 males, with a mean age of 37.41 (±20.56) and 33.87 (±19.58), respectively, there was no statistically significant difference in age between the sexes. (p=0.232; Student’s t-test)”.<br><br>c)Reply to issue no. 4: “Besides forensic identification, please add some other reasons why facial measurements are necessary”<br><br>In the "Discussion" section, we have added the following sentence (with reference) to better explain some other reasons why facial measurements are necessary: “Face measurements are also made for various health purposes, such as in the field of dental prostheses, cosmetic surgery, orthodontics and face masks. In particular, face analysis and proportions have been shown to be important for facial plastic surgeons to evaluate the face during the planning stages of facial reconstructive and cosmetic surgery<br><br>- Shahbazi Z, Ardalani H, Maleki M. Aesthetics of Numerical Proportions in Human Cosmetic Surgery. World J Plast Surg. 2019 Jan;8(1):78-84.<br><br>d)Reply to issue no. 5: “Why not to compare the finding of CT scan with direct measurement of some subjects to verify whether the measurement is affected by the magnification factor or not?”<br><br>Good observation, this study is in anticipation. In the conclusions section we added this sentence: “In the future, studies could be conducted to compare the measurements made on CT scans with those performed directly on the skulls and verify if there is an anthropometric variability that could make the results different”<br><br>For details we invite You to check the file revised and updated in attachment.<br>We really hope that the comments raised by the Reviewers have been satisfied in this manner.<br>We look forward to hearing from You.<br>Sincerely,<br><br>Dr. Francesco De Micco, M.D., Ph.D<br>Corresponding author
**Additional Information:**	
**Question**	**Response**
**Financial Disclosure**<br><br>Enter a financial disclosure statement that describes the sources of funding for the	The author(s) received no specific funding for this work

Powered by Editorial Manager® and ProduXion Manager® from Aries Systems Corporation

work included in this submission. Review the submission guidelines for detailed requirements. View published research articles from *PLOS ONE* for specific examples.<br><br>This statement is required for submission and **will appear in the published article** if the submission is accepted. Please make sure it is accurate.<br><br>**Unfunded studies**<br>Enter: *The author(s) received no specific funding for this work.*<br><br>**Funded studies**<br>Enter a statement with the following details:<br>• Initials of the authors who received each award<br>• Grant numbers awarded to each author<br>• The full name of each funder<br>• URL of each funder website<br>• Did the sponsors or funders play any role in the study design, data collection and analysis, decision to publish, or preparation of the manuscript?<br>• **NO** - Include this sentence at the end of your statement: *The funders had no role in study design, data collection and analysis, decision to publish, or preparation of the manuscript.*<br>• **YES** - Specify the role(s) played.<br><br>* typeset	
**Competing Interests**<br><br>Use the instructions below to enter a competing interest statement for this submission. On behalf of all authors, disclose any competing interests that could be perceived to bias this work—acknowledging all financial support and any other relevant financial or non-financial competing interests.<br><br>This statement is required for submission and **will appear in the published article** if the submission is accepted. Please make sure it is accurate and that any funding sources listed in your Funding Information	The authors have declared that no competing interests exist.

later in the submission form are also declared in your Financial Disclosure statement.<br><br>View published research articles from *PLOS ONE* for specific examples.<br><br>**NO authors have competing interests**<br><br>Enter: *The authors have declared that no competing interests exist.*<br><br>**Authors with competing interests**<br><br>Enter competing interest details beginning with this statement:<br><br>*I have read the journal's policy and the authors of this manuscript have the following competing interests: [insert competing interests here]*<br><br>* typeset	
**Ethics Statement**<br><br>Enter an ethics statement for this submission. This statement is required if the study involved:<br><br>• Human participants<br>• Human specimens or tissue<br>• Vertebrate animals or cephalopods<br>• Vertebrate embryos or tissues<br>• Field research<br><br>Write "N/A" if the submission does not require an ethics statement.<br><br>General guidance is provided below. Consult the submission guidelines for detailed instructions. **Make sure that all information entered here is included in the Methods section of the manuscript.**	Attachment Statement of the Ethics Committee.

**Format for specific study types**<br><br>**Human Subject Research (involving human participants and/or tissue)**<br>• Give the name of the institutional review board or ethics committee that approved the study<br>• Include the approval number and/or a statement indicating approval of this research<br>• Indicate the form of consent obtained (written/oral) or the reason that consent was not obtained (e.g. the data were analyzed anonymously)<br><br>**Animal Research (involving vertebrate animals, embryos or tissues)**<br>• Provide the name of the Institutional Animal Care and Use Committee (IACUC) or other relevant ethics board that reviewed the study protocol, and indicate whether they approved this research or granted a formal waiver of ethical approval<br>• Include an approval number if one was obtained<br>• If the study involved *non-human primates*, add *additional details* about animal welfare and steps taken to ameliorate suffering<br>• If anesthesia, euthanasia, or any kind of animal sacrifice is part of the study, include briefly which substances and/or methods were applied<br><br>**Field Research**<br><br>Include the following details if this study involves the collection of plant, animal, or other materials from a natural setting:<br>• Field permit number<br>• Name of the institution or relevant body that granted permission	
**Data Availability**<br><br>Authors are required to make all data underlying the findings described fully available, without restriction, and from the time of publication. PLOS allows rare exceptions to address legal and ethical concerns. See the PLOS Data Policy and FAQ for detailed information.	Yes - all data are fully available without restriction

A Data Availability Statement describing where the data can be found is required at submission. Your answers to this question constitute the Data Availability Statement and **will be published in the article**, if accepted.<br><br>**Important:** Stating 'data available on request from the author' is not sufficient. If your data are only available upon request, select 'No' for the first question and explain your exceptional situation in the text box.<br><br>Do the authors confirm that all data underlying the findings described in their manuscript are fully available without restriction?	
**Describe where the data may be found in full sentences. If you are copying our sample text, replace any instances of XXX with the appropriate details.**<br><br>• If the data are **held or will be held in a public repository**, include URLs, accession numbers or DOIs. If this information will only be available after acceptance, indicate this by ticking the box below. For example: *All XXX files are available from the XXX database (accession number(s) XXX, XXX.*).<br>• If the data are all contained **within the manuscript and/or Supporting Information files**, enter the following: *All relevant data are within the manuscript and its Supporting Information files.*<br>• If neither of these applies but you are able to provide **details of access elsewhere**, with or without limitations, please do so. For example:<br><br>*Data cannot be shared publicly because of [XXX]. Data are available from the XXX Institutional Data Access / Ethics Committee (contact via XXX) for researchers who meet the criteria for access to confidential data.*<br><br>*The data underlying the results presented in the study are available from (include the name of the third party*	All relevant data are within the manuscript

and contact information or URL).<br>• This text is appropriate if the data are owned by a third party and authors do not have permission to share the data.<br><br>* typeset	
Additional data availability information:	

Powered by Editorial Manager® and ProduXion Manager® from Aries Systems Corporation

Manuscript
Click here to access/download;Manuscript;Manuscript.docx

Anthropometric analysis of orbital and nasal parameters for sexual dimorphism: new anatomical evidences in the field of personal identification

New anatomical evidences for forensic personal identification

Roberto Scendoni [1], Jeta Kelmendi [2], Isabella Lima Arrais Ribeiro [3], Mariano Cingolani [1], Francesco De Micco [4 *], Roberto Cameriere [5]

[1] Department of Law, Institute of Legal Medicine (AgEstimation Project), University of Macerata, Macerata, Italy
[2] Faculty of Medicine, Department of Orthodontic, University of Prishtina, Kosovo. Alma Mater Europaea, Campus Rezonanca, Prishtina, Kosovo
[3] Postgraduate Program in Dentistry, Federal University of Paraíba, Campus I, João Pessoa, PB, 58051900, Brazil
[4] Bioethics and Humanities Research Unit, Campus Bio-Medico University of Rome, 00128 Rome, Italy
[5] Department of Forensic Medicine (AgEstimation Project), University of Molise, Italy

*Corresponding author
E-mail: f.demicco@policlinicocampus.it (FDM)

Abstract

Orbital and nasal parameters among modern humans show considerable variation, which affects facial shape, and these characteristics vary according to race, region, and period in evolution. The aim of the study was to ascertain whether there are sex differences in the orbital and/or nasal indexes (OI and NI) and/or the single measurements used to calculate these in a Kosovar population. The following parameters were taken into consideration: orbital height (OH), orbital width (OW), nasal height (NH), and nasal width (NW). The ratios between orbital index/nasal index (RONI) were calculated. All measurements were obtained from a population sample comprising 408 individuals. The sample was randomly divided into two subsamples, one constituting approximately 2/3 (n=251) of the initial sample, and the other containing approximately 1/3 of the initial sample (n=157). This division was important for the steps of training (2/3 of sample) and testing (validation) (1/3 of sample) variables of interest for the prediction of sex. Therefore, 251 individuals were evaluated, 110 females and 141 males, with a mean age of 37.41 (±20.56) and 33.87 (±19.58), respectively. The data were collected from head CT scans using digital calipers and statistically analyzed (Kolmogorov-Smirnov normality test, Levene's test, Student's t-test and logistic regression). After prediction analysis, the cutoff points were selected: 36.76 for NW measurements, and 50.00 for NH measurements. For two variables, measurements below these cutoff values indicated female sex and measurements above indicated male sex. The accuracy in sex prediction was 52.86% (CI95% = 45.05% - 60.67%) for NW and 64.96% for NH (CI95% = 57.50% – 72.42%). The difference between male and female indexes was statistically significant ($P < 0.05$). The anthropometric study revealed that only NW and NH are configured as predictors of sexual dimorphism. It could be useful to increase the number of samples to test the discriminant function in other population groups.

Introduction

Assessing ethnicity and identifying skeletal remains are important and difficult tasks for forensic scientists. Numerous methods using craniofacial measurements and indexes are available and useful for these purposes. The manner of determining the parameters needed for the estimation of these directories depends on the sort of samples used [1]. Extensive research has been conducted

worldwide on the dimensions and volumes of the orbits and nasal cavity. Calipers are still commonly used as linear measuring instruments to measure the dimensions of the orbital and nasal margins [2, 3]. However, advanced radiological techniques such as computed tomography (CT) scanners have proven to be a better choice since they facilitate sample collection [2]. The orbital index (OI) and nasal index (NI) are calculated using measurements between several landmarks in the respective facial cavity [3]. Orbital and nasal cavity dimensions and volumes, in various populations, have been investigated by many authors [4-6], and different populations have been found to exhibit considerable variation in these values [7-10]. Indeed, population-based variations are predictable results of evolutionary processes; natural selection acts on inheritable mutations, which may relate to current environmental pressures, genetic implication, past and present hybridization between geographically distinct populations, and the present selective adaptation of human varieties to their environment.

Overall, these studies have indicated some level of ethnic and racial variation in the OI and NI of various population groups. In modern human groups, the appearance of the orbital and nasal cavities varies considerably [5]. Craniofacial morphometric analysis is conducted using OI and NI parameters, based on the ratio of the orbital/nasal height to its width multiplied by 100. These ratios affect the shape of the face and vary with race, regions within the same race, and periods in evolution [11-14].

However, to date there has been no morphometric study on OI and NI and their relationship to biological sex prediction in the Kosovar population. Therefore, this study of the orbital and nasal morphometry in the skulls of a Kosovar population using a CT scanner represents an important contribution to the literature which will expand knowledge in various fields, such as forensic medicine, and help to explore trends in evolutionary and ethnic differences, among different races in particular, where forensic data is not available. Thus, the main objective of this research is to ascertain whether there are sex differences in the orbital and/or nasal indexes and/or the single measures used to calculate these.

Materials and Methods

A retrospective study design was used in this research involving the Kosovar ethnic group living in Kosovo. Inclusion criteria were Kosovar males and females over the age of 18, who underwent

CT scan to investigate neurological symptoms. Exclusion criteria included growth diseases, endocrine disorders or osteodystrophy, previous fractures of the of the skull and the facial massif. Finally, all blurred TC images were disregarded. The CT scans were anonymously extracted and limited data were extracted that were strictly necessary and relevant for the conduct of the study. The extracted data are all available in the manuscript. A sample of 408 individuals was randomly divided into two subsamples, one constituting approximately 2/3 (n=251) of the initial sample, and the other containing approximately 1/3 of the initial sample (n=157). This division was important for the steps of training (2/3 of sample) and testing (validation) (1/3 of sample) variables of interest for the prediction of sex. Therefore, 251 individuals were evaluated, 110 females and 141 males, with a mean age of 37.41 (±20.56) and 33.87 (±19.58), respectively. The sample of the present study was calculated based on the effects observed in the study by Kotian et al. [15]. From this study, the mean effect size of difference between groups of 0.20 (Cohen's d) was observed. Adopting a 5% type I error (95% study confidence level), a 20% type II error (80% study power), the effect size observed in the mentioned study and, considering the comparison tests of the groups of the bilateral type (two-sided), it was calculated that at least 394 sample units were necessary for the accomplishment of the study. As they are available, we conducted the study with 408 sample units. The calculations were performed using the G*Power statistical software.

The study was carried out on frontal views of head CT scans performed on patients who had attended radiology units (private and public) in various cities in Kosovo. The height and width of the orbital cavity and nasal aperture were measured on the CT scan of the frontal bone using a digital ruler (in mm). The following parameters were measured: orbital height (OH) and nasal height (NH) – the maximum distance between the upper and lower margins of the orbital cavity rim and nasal aperture rim (Fig 1); orbital width (OW) and nasal width (NW) – the distance from the midpoint of the medial margin of the orbital/nasal aperture to the midpoint of the lateral margin of the same orbit/nasal aperture. The mean orbital and nasal indexes were calculated by the formula: height/width x 100. Only normal CT scans were used (cases with bone pathology and fractures were excluded from the study). Finally, the ratios between orbital index/nasal index (RONI) were calculated.

Fig. 1. Plain film showing the orbital and nasal margins and measurements of orbital/nasal width and height.

The data were analyzed in R software (version 3.6.3, R Core Team, R Foundation for Statistical Computing, Vienna, Austria). Initially, the data were tested for normal distribution, verifying a normal distribution for all the quantitative variables ($p>0.05$; Kolmogorov-Smirnov normality test). Sequentially, the values of the variables OH, OW, NW, NH, OI, NI, and RONI were compared between male and female subjects (Levene's test and Student's t-test), and dependence of outcome (sex prediction) on each variable of interest was evaluated by logistic regression, using female sex as the reference group. The female gender was chosen as the reference category because the measures of interest were lower for females and leaving this category as a reference makes the interpretation of the odds ratio findings more understandable. Receiver operating characteristic curves (ROCs) (Figs 2 and 3) were generated for the NW and NH variables, these being the most accurate predictors of sex. The best cutoff points for each variable of interest were obtained from the equation "$Ir.eta=1/1+e^{-(\alpha+\beta X)}$": 36.76 for NW measurements, and 50.00 for NH measurements. For the two variables under study, values below the cutoff score were interpreted as indicating female sex, while values above the cutoff score were taken to indicate male sex. In the second phase of this study, these cutoff points were tested in an independent sample of 157 other individuals. A contingency table (confusion matrix) was generated, along with the respective predictive values of sensitivity and specificity (Se and Sp), positive and negative predictive values (PV+ and PV-), the positive and negative likelihood ratios (LR+ and LR-), and accuracy (Acc), including their respective confidence intervals 95% (CI95%). The significance level was 5% for all analyses. The Ethical Issues Committee of the Kosovo Chamber of Dentists approved the study (nr. 27, 18/05/2022).

Fig 2. Receiver operating characteristic curve for prediction analysis of sex from NW

Fig 3. Receiver operating characteristic curve for prediction analysis of sex from NH.

Results

251 individuals were evaluated, 110 females and 141 males, with a mean age of 37.41 (±20.56) and 33.87 (±19.58), respectively, there was no statistically significant difference in age between the sexes. ($p=0.232$; Student's t-test).

Table 1 presents the distributions and comparisons of measurements for males and females.

Table 2 shows the results of the analysis for each predictor variable. It was not possible to fit a multiple model, with more than one variable being a predictor together with another, among the variables evaluated. For this reason, the univariate analysis is shown in Table 2, where it is observed that, in isolation, the variables NW and NH were configured as predictors of sex. For this reason, Table 2 describes only the univariate analysis.

In the data validation step ($n=157$), the accuracy of sex prediction was 52.86% for NW and 64.96% for NH, considering the test cutoff points indicated (Table 3).

Table 3 describes the values obtained for the sample used in the test phase of the new cutoff points, which were applied to the test sample, generating the values of sensitivity (Se), specificity (Sp), positive predictive value (PV+), negative predictive value (PV-), positive likelihood (LR+), negative likelihood (LR-), and accuracy (Acc).

For NW and NH, moderate accuracies were obtained. Positive and negative predictive values were moderate and high, respectively, indicating moderate and high probabilities of correct classification, for females and males, respectively.

Table 3 also shows the positive and negative predictive values (LR+ and LR-) of the NH and NW variables, that is the probability that subjects classified by the model as male truly are male, and the probability that subjects classified by the model as female truly are female.

Table 1. Distribution of measurements of OH, OW, NW, NH, OI, NI, and RONI, and comparison between males and females

Variable	Predict categories	Measurement value Mean (SD)	p-value
**OH**	Female	38.42 (±5.14)	0.129
	Male	39.44 (±5.32)	
**OW**	Female	41.54 (±4.01)	0.252
	Male	42.15 (±4.36)	
**NW**	Female	34.15 (±5.07)	0.030
	Male	35.66 (±5.70)	
**NH**	Female	49.14 (±7.36)	0.028
	Male	51.25 (±7.59)	
**OI**	Female	0.92 (±0.09)	0.363
	Male	0.93 (±0.10)	
**NI**	Female	0.70 (±0.09)	0.984
	Male	0.70 (±0.09)	
**RONI**	Female	1.33 (±0.20)	0.453
	Male	1.36 (±0.23)	

Legend: OH (orbital height); OW (orbital width); NW (nasal width); NH (nasal height); OI (orbital index = OH/OW); NI (nasal index = NW/NH); RONI (OI/NI). Student's t-test. Significance level = 5%.

Table 2. Results of sex prediction from OH, OW, NW, NH, OI, NI, and RONI

Variable	Sex	p-value	OR (CI 95%)
**OH**	Female	0.130	1
	Male		1.03 (0.98 - 1.08)
**OW**	Female	0.252	1
	Male		1.03 (1.02 - 1.10)
**NW**	Female	0.031	1
	Male		1.05 (1.01 - 1.08)
**NH**	Female	0.030	1
	Male		1.03 (1.01 - 1.05)
**OI**	Female	0.376	1
	Male		3.12 (0.61 - 5.62)
**NI**	Female	0.989	1
	Male		0.98 (-1.56 - 3.53)
**RONI**	Female	0.462	1
	Male		1.53 (0.39 – 2.66)

Legend: OH (orbital height); OW (orbital width); NW (nasal width); NH (nasal height); OI (orbital index = OH/OW); NI (nasal index = NW/NH); RONI (OI/NI). Logistic regression. OR=Odds ratio. IC 95% = Confidence interval 95%. Significance level = 5%.

Table 3. Contingency table and predictive values for classification of individuals based on NW and NH measurements

Variable		Real classification		Se	Sp	PV+	PV-	LR+	LR-	Acc
		Female	Male							
NW classification	Female	43	58	72.88% (65.93-79.83)	40.81% (33.12-48.50)	42.57% (34.84-50.30)	71.42% (64.35-78.49)	1.23 (0.54-1.92)	0.66 (0.58-0.73)	52.86% (45.05-60.67)
	Male	16	40							
NH classification	Female	33	29	55.93% (48.16-63.70)	70.40% (63.26-77.54)	53.22% (45.41-61.03)	72.63% (65.66-79.60)	1.88 (1.05-2.71)	0.62 (0.54-0.69)	64.96% (57.50-72.42)
	Male	26	69							

Legend: Se = Sensitivity; Sp = Specificity; PV+ = Positive predictive value; PV- = Negative predictive value; LR+ = Positive likelihood ratio; LR- = Negative likelihood ratio; Acc=Accuracy.

Discussion

Human beings are unique in terms of their physical growth and development, which means that related measurements can be used to identify individuals based on physical variability. The morphological features of the skull are useful in the identification of dead and living persons [16, 17]. Measurements of facial anatomy, including sinuses, nasal cavity, or orbital cavity, are now widely used in forensic identification, even if it is necessary to carefully differentiate between different ethnic groups. Face measurements are also made for various health purposes, such as in the field of dental prostheses, cosmetic surgery, orthodontics and face masks. In particular, face analysis and proportions have been shown to be important for facial plastic surgeons to evaluate the face during the planning stages of facial reconstructive and cosmetic surgery [18].

Craniometric parameters including NI and OI have been investigated in prior research to estimate sex in forensic medicine. Preliminary knowledge of these measurements is essential for correct application since they vary from one population to another. The literature shows that there are significant differences in orbital and nasal morphometry between individuals in relation to age, sex, and ethnicity [19].

This study was conducted to establish the orbital and nasal indexes of Kosovar adults using skull CT scans. Previous studies have shown that measurements for the dimensions of orbital and nasal cavities taken from human skulls are similar to those obtained by CT scans [20], and the

consistency of measurements using CT images has been evaluated in the last decade [21]. Our analysis was performed on head CT scans of patients with headache disorders, neurological deficits (e.g., poststroke), epilepsy, and other medical issues. This research excluded individuals with a history of bone pathology, and the sample can therefore be considered a “normal population” [22].

The human nose, as the most protruding part of the face, is variable in size and shape [23, 24]. Divergence of nose shape and prominence can be explained by ethnic influences and environmental and climatic conditions [23-25]. In the present study using CT scans, the measurements of both the nasal aperture and orbital cavity were higher in males compared to females. These observations are in agreement with the results of Vidya et al., who examined dry skulls of South Indian origin [25], and with those of Nasir et al., who studied nasal indexes across four Indian states [26]. Similarly, the nasal index, calculated from height and weight measurements of the nasal cavity, was found to be higher in males than in females in this research, in line with the findings of Staka et al. (on another Kosovar population), Hegazy (on Egyptians), Sforza et al., and Franciscus and Long [12, 13, 27, 28]. Our results also showed that Kosovar adult males have a mean orbital index of 0.93 (0.10), while the equivalent score for adult females is 0.92 (0.09) (Table 1). Results from this study concur with previous research that found a significant sex difference ($p<0.05$) in the orbital indexes of Indian and Nigerian adults [29]. The present study does not support other investigations that have noted a higher OI in females than in males. The differences seen in these populations may be attributed to the different age groups of subjects across various studies [30]. Also, in our study the mean orbital height and weight measurements for females and males are half of those recorded in Indian, Nigerian, Malawian, Bini, and Urhobo populations [31]. We found that there was a significant difference in the orbital index between males and females, mainly because males exhibit wider orbital cavities than females. Some authors have reported differences in the volume of OI between males and females, whereas others have shown no such difference [32-35].

Lusted and Keats reported that orbital parameters obtained from radiographs are slightly different from those obtained from direct measurement of human skulls and that this difference could be attributed to the magnification factor of X-ray machines [35]. Indeed, the results of the present study might have been affected by the magnification factor and therefore may not present completely true measurements of the orbital or nasal cavity. Other differences between our

findings and those of other authors could be due to different patterns of craniofacial growth caused by racial, ethnic, social, environmental, and nutritional factors [28, 36, 37].

Differences in nose shape and appearance are influenced by ethnic factors and environmental climatic conditions. A study on the Dayak Kenyah population in North Kalimantan, Indonesia, found that the predominant nasal shape was mesorrhine and attributed this to environmental factors, concluding that differences between nasal shapes may be used to trace geographic origins [38]. In a study on a South Indian population, using MDCT 2D scans to measure nasal index and nasal parameters, Kotian et al. found that the measurements of nasal aperture were greater in males compared to females [15]; these findings are in agreement with the present study. Regardless of sexual dimorphism, it is predictable that ethnic groups in the same climatic regions should have similarities in their nasal indexes. In any case, the point that is most relevant for forensic investigations is that anthropometric data of the face and nose would be useful for sex determination, and Svitlana and Themozhi have suggested that nasal proportions are indeed a useful anthropometrical tool in determination of sex in forensic science and classification of fossil remains [39].

This study indicates that NW and NH have potential to classify individuals as female and male with predictive accuracy of around 52.86% and 64.96%, respectively. Anthropological studies agree that variations in the nasal index are related to the climate. Variation in the bony nasal cavity probably stems from man’s ability to adapt to the environment. The peculiarity of this study is the fact that a different predictivity in the estimation of sex, with statistical significance, lies not in the calculation of the NI, but in the individual measurements which, if compared, form the NI (NW and NH). In particular, females tend to have a lower NW than that of males, as well as a higher NH. However, it should be noted that, as limitation, the study has moderate accuracy with not very high power to classify sex. In a larger pool of samples or in other Caucasian populations we could find even higher levels of accuracy.

To understand whether this nasal anatomical variation is due to the adaptation of the Kosovar population to the specific environment or whether it concerns other ethnic groups, broadly speaking, it would be useful to extend the analysis to other neighboring and non-neighboring populations.

Conclusion

Sex estimation is essential for completing an individual's biological profile and therefore constitutes an important part of the human identification process in the forensic field. It also has significant ethical and legal implications [40-42].

The current anthropometric study found that the metric parameters necessary for the calculation of NI showed pronounced sexual dimorphism in a large Kosovar population. Since the equation developed in the stepwise discriminant analysis was intended for this specific population, it may produce different results for other population groups. Further studies are recommended to evaluate and characterize orbital and nasal parameters among different populations; it could be useful test the discriminant function in populations from different countries, and study the dependence of anthropometric data on sex and age. Therefore, conducting a comparative study between different groups would be very interesting in the field of personal identification for forensic purposes. In the future, studies could be conducted to compare the measurements made on CT scans with those performed directly on the skulls and verify if there is an anthropometric variability that could make the results different.

Acknowledgments

Thanks to Jemma Dunnill for proofreading the manuscript.

References

1. Catalina-Herrera CJ. Morphometric study of the orbit's base in male and female skulls of Spaniards. Bull Assoc Anat (Nancy). 1988;72(217-218):5-7.
2. Giles E, Elliot O. Race identification from cranial measurements. J Forensc Sci. 1962; 7: 147-57.
3. Nitek S, Wysocki J, Reymond J, Piasecki K. Correlations between selected parameters of the human skull and orbit. Med Sci Monit. 2009;15(12):BR370-BR377.

4. Forbes G, Gehring DG, Gorman CA, Brennan MD, Jackson IT. Volume measurements of normal orbital structures by computed tomographic analysis. AJR Am J Roentgenol. 1985;145(1):149-154. doi:10.2214/ajr.145.1.149
5. Karampatakis V, Natsis K, Gigis P, Stangos NT. Orbital depth measurements of human skulls in relation to retrobulbar anesthesia. Eur J Ophthalmol. 1998;8(2):118-120.
6. Ezeuko VC, Om'Iniabohs FAE. Radiologic evaluation of the orbital index among the Igbo ethnic group of Nigeria. Eur J Anat. 2015; 19: 9-14.
7. Dilmen G, Köktener A, Turhan NO, Tez S. Growth of the fetal lens and orbit. Int J Gynaecol Obstet. 2002;76(3):267-271. doi:10.1016/s0020-7292(01)00581-1
8. Merz E, Bahlmann F, Weber G. Volume scanning in the evaluation of fetal malformations: a new dimension in prenatal diagnosis. Ultrasound Obstet Gynecol. 1995;5(4):222-227. doi:10.1046/j.1469-0705.1995.05040222.x
9. Rajangam S, Kulkarni RN, Quadrilos L, Sreenivasulu S. Orbital dimensions. Indian J Anat. 2012; 1: 5-9.
10. Mehta M, Saini V, Nath S, Patel NM, Menon SK. CT scan images to determine the origin from craniofacial indices for Gujarati population. Journal of Forensic Radiology and Imaging. 2014; 2: 64-71.
11. Oladipo GS, Olabiyi AO, Oremosu AA, NoronhaCC. Nasal indices among major ethnic groups in Southern Nigeria. SRE. 2007; 2(1): 20-22.
12. Staka G, Dragidella F, Disha M. Anthropometric study of nasal index of the Kosovo Albanian population. Online Journal of Anthropology. 2012; 8: 457-62.
13. Hegazy AA. Anthropometric study of nasal index of Egyptians. Int J Anat Res. 2014; 2: 761-7.
14. Ravichandran S, Yuvraj Babu K, Mohanraj KG. Correlation of facial and nasal index in gender determination. Drug Invention Today. 2018; 10(12).
15. Kotian R, Bakkannavar SM, Shekhar H, Pradhan P, Nayak VC. Sex determination based on nasal index and nasal parameters using (big bore 16 slice) multidetector computed tomography 2D scans. Indian J Forensic Community Med. 2015; 2:167-71.
16. Hsiao TH, Chang HP, Liu KM. Sex determination by discriminant function analysis of lateral radiographic cephalometry. J Forensic Sci. 1996;41(5):792-795.

17. Cameriere R, Zolotenkova GV, Kuznetsov IA, Scendoni R, Pigolkin YI. Sex estimation based on the anthropometric measurements of thyroid cartilage using discriminant analysis. Egypt J Forensic. 2021; Sci. 11.
18. Shahbazi Z, Ardalani H, Maleki M. Aesthetics of Numerical Proportions in Human Cosmetic Surgery. World J Plast Surg. 2019 ; 8(1):78-84.
19. Kanjani V, Rani A, Kanjani D. Morphometric analysis of the orbital aperture in north indian population: a retrospective digital forensic study. Int J Appl Basic Med Res. 2019;9(2):85-88. doi:10.4103/ijabmr.IJABMR_404_18
20. Patil KR, Mody RN. Determination of sex by discriminant function analysis and stature by regression analysis: a lateral cephalometric study. Forensic Sci Int. 2005;147(2-3):175-180. doi:10.1016/j.forsciint.2004.09.071
21. Krishan K. Estimation of stature from cephalo-facial anthropometry in north Indian population. Forensic Sci Int. 2008;181(1-3):52.e1-52.e526. doi:10.1016/j.forsciint.2008.08.0
22. Veyre-Goulet SA, Mercier C, Robin O, Guérin C. Recent human sexual dimorphism study using cephalometric plots on lateral teleradiography and discriminant function analysis. J Forensic Sci. 2008;53(4):786-789. doi:10.1111/j.1556-4029.2008.00759.x
23. Hsiao TH, Tsai SM, Chou ST, Pan JY, Tseng YC, Chang HP, et al. Sex determination using discriminant function analysis in children and adolescents: a lateral cephalometric study. Int J Legal Med. 2010;124(2):155-160. doi:10.1007/s00414-009-0412-1
24. Naikmasur VG, Shrivastava R, Mutalik S. Determination of sex in South Indians and immigrant Tibetans from cephalometric analysis and discriminant functions. Forensic Sci Int. 2010;197(1-3):122.e1-122.e1226. doi:10.1016/j.forsciint.2009.12.052
25. Vidya CS, Shamasundar NM, Manjunatha B, Raichurkar K. Evaluation of size and volume of maxillary sinus to determine sex by 3D computerized tomography scan method using dry skulls of South Indian origin. International Journal of Current Research and Review. 2013; 5: 97.
26. Nasir N, Asad MR, Muzammil K, Hassan A, Alshalwi T, Khan MR. Anthropometric study of nasal indices in four Indian Stat. Cli. Pract. 2021; 18: 1620-5.
27. Sforza C, Dellavia C, Colombo A, Serrao G, Ferrario VF. Nasal dimensions in normal subjects: conventional anthropometry versus computerized anthropometry. Am J Med Genet A. 2004; 130: 228-33. doi: 10.1002/ajmg.a.30275. PMID: 15378539.

28. Franciscus RG, Long JC. Variation in human nasal height and breadth. Am J Phys Anthropol. 1991;85(4):419-427. doi:10.1002/ajpa.1330850406
29. Oladipo GS, Eroje MA, Fahwehinmi HB. Anthropometric comparison of nasal indices between Andoni and Okrika tribes of Rivers State, Nigeria. International Journal of Medicine and Medical Sciences 2009; 1: 135-7.
30. Ahmed AA, Mohammed H, Mohamed H. Sex determination from cranial measurements in recent northern Sudanese. Khartoum Med J; 2011; 1: 539–47
31. O'Donnell C, Iino M, Mansharan K, Leditscke J, Woodford N. Contribution of postmortem multidetector CT scanning to identification of the deceased in a mass disaster: Experience gained from the 2009 Victorian bushfires. Forensic Sci Int. 2011;205(1-3):15-28. doi:10.1016/j.forsciint.2010.05.026.
32. Amin MF, Hassan EI. Sex identification in Egyptian population using Multidetector Computed Tomography of the maxillary sinus. J Forensic Leg Med. 2012;19(2):65-69. doi:10.1016/j.jflm.2011.10.005
33. Sukumar S, Yadav S, Vipinkumar G. Sex determination by mastiod process in South Indian population by 3D-computer tomography imaging. Int J PharmBiol Sci. 2012; 4:193–5.
34. de Froidmont S, Grabherr S, Vaucher P, De Cesare M, Egger C, Papageorgopoulou C, et al. Virtual anthropology: a comparison between the performance of conventional X-ray and MDCT in investigating the trabecular structure of long bones. Forensic Sci Int. 2013;225(1-3):53-59. doi:10.1016/j.forsciint.2012.10.029
35. Lusted LB, Keats ET. Geometric distortion of roentgen image and its correction. In: Keats TE, editor. Atlas of roentgenographic measurements, Chicago Year Book Medical Pub; 1977. pp. 1-4.
36. Lusted LB, Keats ET. Geometric distortion of roentgen image and its correction. In: Keats TE, editor. Atlas of roentgenographic measurements, Chicago Year Book Medical Pub; 1977. p. 30
37. Howale D, Pradhan R, Jain L, Lekharu R. The calculation of various craniofacial indices in Maharastra population. Int J Curr Res; 2012; 4: 162–6.
38. Marini MI, Angrosidy H, Kurniawan A, Margareth MS. The anthropological analysis of the nasal morphology of Dayak Kenyah population in Indonesia as a basic data for forensic identification. Translational Research in Anatomy. 2020; 19.

39. Svitlana AS, Themozhi MS. Study of nasal index-a review. IJARIIE. 2019; 5: 110-5.
40. De Micco F, Martino F, Campobasso CP. Ethical issues in age assessment by the third molar development. Australian Journal of Forensic Sciences. 2022; 54:88-99.
41. Angelakopoulos N, Galić I, De Luca S, Campobasso CP, Martino F, De Micco F, et al.. Skeletal age assessment by measuring planar projections of carpals and distal epiphyses of ulna and radius bones in a sample of South African subadults, Australian Journal of Forensic Sciences, 2022; 54:1.DOI: 10.1080/00450618.2020.1766111
42. Bakkannavar SM., Manjunath S, Nayak VC, Pradeep Kumar G. Canine index – A tool for sex determination, Egyptian Journal of Forensic Sciences, Volume 5, Issue 4, 2015, 157-161

Manuscript Click here to access/download;Manuscript;Revised Manuscript.docx

Anthropometric analysis of orbital and nasal parameters for sexual dimorphism: new anatomical evidences in the field of personal identification

New anatomical evidences for forensic personal identification

Roberto Scendoni [1], Jeta Kelmendi [2], Isabella Lima Arrais Ribeiro [3], Mariano Cingolani [1], Francesco De Micco [4*], Roberto Cameriere [5]

[1] Department of Law, Institute of Legal Medicine (AgEstimation Project), University of Macerata, Macerata, Italy
[2] Faculty of Medicine, Department of Orthodontic, University of Prishtina, Kosovo. Alma Mater Europaea, Campus Rezonanca, Prishtina, Kosovo
[3] Postgraduate Program in Dentistry, Federal University of Paraíba, Campus I, João Pessoa, PB, 58051900, Brazil
[4] Bioethics and Humanities Research Unit, Campus Bio-Medico University of Rome, 00128 Rome, Italy
[5] Department of Forensic Medicine (AgEstimation Project), University of Molise, Italy

*Corresponding author
E-mail: f.demicco@policlinicocampus.it (FDM)

Abstract

Orbital and nasal parameters among modern humans show considerable variation, which affects facial shape, and these characteristics vary according to race, region, and period in evolution. The aim of the study was to ascertain whether there are sex differences in the orbital and/or nasal indexes (OI and NI) and/or the single measurements used to calculate these in a Kosovar population. The following parameters were taken into consideration: orbital height (OH), orbital width (OW), nasal height (NH), and nasal width (NW). The ratios between orbital index/nasal index (RONI) were calculated. All measurements were obtained from a population sample comprising 408 individuals. The sample was randomly divided into two subsamples, one constituting approximately 2/3 (n=251) of the initial sample, and the other containing approximately 1/3 of the initial sample (n=157). This division was important for the steps of training (2/3 of sample) and testing (validation) (1/3 of sample) variables of interest for the prediction of sex. Therefore, 251 individuals were evaluated, 110 females and 141 males, with a mean age of 37.41 (±20.56) and 33.87 (±19.58), respectively. The data were collected from head CT scans using digital calipers and statistically analyzed (Kolmogorov-Smirnov normality test, Levene’s test, Student’s t-test and logistic regression). After prediction analysis, the cutoff points were selected: 36.76 for NW measurements, and 50.00 for NH measurements. For two variables, measurements below these cutoff values indicated female sex and measurements above indicated male sex. The accuracy in sex prediction was 52.86% (CI95% = 45.05% - 60.67%) for NW and 64.96% for NH (CI95% = 57.50% – 72.42%). The difference between male and female indexes was statistically significant ($P < 0.05$). The anthropometric study revealed that only NW and NH are configured as predictors of sexual dimorphism. It could be useful to increase the number of samples to test the discriminant function in other population groups.

Introduction

Assessing ethnicity and identifying skeletal remains are important and difficult tasks for forensic scientists. Numerous methods using craniofacial measurements and indexes are available and useful for these purposes. The manner of determining the parameters needed for the estimation of these directories depends on the sort of samples used [1]. Extensive research has been conducted

worldwide on the dimensions and volumes of the orbits and nasal cavity. Calipers are still commonly used as linear measuring instruments to measure the dimensions of the orbital and nasal margins [2, 3]. However, advanced radiological techniques such as computed tomography (CT) scanners have proven to be a better choice since they facilitate sample collection [2]. The orbital index (OI) and nasal index (NI) are calculated using measurements between several landmarks in the respective facial cavity [3]. Orbital and nasal cavity dimensions and volumes, in various populations, have been investigated by many authors [4-6], and different populations have been found to exhibit considerable variation in these values [7-10]. Indeed, population-based variations are predictable results of evolutionary processes; natural selection acts on inheritable mutations, which may relate to current environmental pressures, genetic implication, past and present hybridization between geographically distinct populations, and the present selective adaptation of human varieties to their environment.

Overall, these studies have indicated some level of ethnic and racial variation in the OI and NI of various population groups. In modern human groups, the appearance of the orbital and nasal cavities varies considerably [5]. Craniofacial morphometric analysis is conducted using OI and NI parameters, based on the ratio of the orbital/nasal height to its width multiplied by 100. These ratios affect the shape of the face and vary with race, regions within the same race, and periods in evolution [11-14].

However, to date there has been no morphometric study on OI and NI and their relationship to biological sex prediction in the Kosovar population. Therefore, this study of the orbital and nasal morphometry in the skulls of a Kosovar population using a CT scanner represents an important contribution to the literature which will expand knowledge in various fields, such as forensic medicine, and help to explore trends in evolutionary and ethnic differences, among different races in particular, where forensic data is not available. Thus, the main objective of this research is to ascertain whether there are sex differences in the orbital and/or nasal indexes and/or the single measures used to calculate these.

Materials and Methods

A retrospective study design was used in this research involving the Kosovar ethnic group living in Kosovo. Inclusion criteria were Kosovar males and females over the age of 18, who underwent

CT scan to investigate neurological symptoms. Exclusion criteria included growth diseases, endocrine disorders or osteodystrophy, previous fractures of the of the skull and the facial massif. Finally, all blurred TC images were disregarded. The CT scans were anonymously extracted and limited data were extracted that were strictly necessary and relevant for the conduct of the study. The extracted data are all available in the manuscript. A sample of 408 individuals was randomly divided into two subsamples, one constituting approximately 2/3 (n=251) of the initial sample, and the other containing approximately 1/3 of the initial sample (n=157). This division was important for the steps of training (2/3 of sample) and testing (validation) (1/3 of sample) variables of interest for the prediction of sex. Therefore, 251 individuals were evaluated, 110 females and 141 males, with a mean age of 37.41 (±20.56) and 33.87 (±19.58), respectively. The sample of the present study was calculated based on the effects observed in the study by Kotian et al. [15]. From this study, the mean effect size of difference between groups of 0.20 (Cohen's d) was observed. Adopting a 5% type I error (95% study confidence level), a 20% type II error (80% study power), the effect size observed in the mentioned study and, considering the comparison tests of the groups of the bilateral type (two-sided), it was calculated that at least 394 sample units were necessary for the accomplishment of the study. As they are available, we conducted the study with 408 sample units. The calculations were performed using the G*Power statistical software.

The study was carried out on frontal views of head CT scans performed on patients who had attended radiology units (private and public) in various cities in Kosovo. The height and width of the orbital cavity and nasal aperture were measured on the CT scan of the frontal bone using a digital ruler (in mm). The following parameters were measured: orbital height (OH) and nasal height (NH) – the maximum distance between the upper and lower margins of the orbital cavity rim and nasal aperture rim (Fig 1); orbital width (OW) and nasal width (NW) – the distance from the midpoint of the medial margin of the orbital/nasal aperture to the midpoint of the lateral margin of the same orbit/nasal aperture. The mean orbital and nasal indexes were calculated by the formula: height/width x 100. Only normal CT scans were used (cases with bone pathology and fractures were excluded from the study). Finally, the ratios between orbital index/nasal index (RONI) were calculated.

Fig. 1. Plain film showing the orbital and nasal margins and measurements of orbital/nasal width and height.

The data were analyzed in R software (version 3.6.3, R Core Team, R Foundation for Statistical Computing, Vienna, Austria). Initially, the data were tested for normal distribution, verifying a normal distribution for all the quantitative variables ($p>0.05$; Kolmogorov-Smirnov normality test). Sequentially, the values of the variables OH, OW, NW, NH, OI, NI, and RONI were compared between male and female subjects (Levene's test and Student's t-test), and dependence of outcome (sex prediction) on each variable of interest was evaluated by logistic regression, using female sex as the reference group. The female gender was chosen as the reference category because the measures of interest were lower for females and leaving this category as a reference makes the interpretation of the odds ratio findings more understandable. Receiver operating characteristic curves (ROCs) (Figs 2 and 3) were generated for the NW and NH variables, these being the most accurate predictors of sex. The best cutoff points for each variable of interest were obtained from the equation "Ir.eta=1/1+$e^{-(\alpha + \beta X)}$": 36.76 for NW measurements, and 50.00 for NH measurements. For the two variables under study, values below the cutoff score were interpreted as indicating female sex, while values above the cutoff score were taken to indicate male sex. In the second phase of this study, these cutoff points were tested in an independent sample of 157 other individuals. A contingency table (confusion matrix) was generated, along with the respective predictive values of sensitivity and specificity (Se and Sp), positive and negative predictive values (PV+ and PV-), the positive and negative likelihood ratios (LR+ and LR-), and accuracy (Acc), including their respective confidence intervals 95% (CI95%). The significance level was 5% for all analyses. The Ethical Issues Committee of the Kosovo Chamber of Dentists approved the study (nr. 27, 18/05/2022).

Fig 2. Receiver operating characteristic curve for prediction analysis of sex from NW

Fig 3. Receiver operating characteristic curve for prediction analysis of sex from NH.

Results

251 individuals were evaluated, 110 females and 141 males, with a mean age of 37.41 (±20.56) and 33.87 (±19.58), respectively, there was no statistically significant difference in age between the sexes. ($p=0.232$; Student's t-test).

Table 1 presents the distributions and comparisons of measurements for males and females.

Table 2 shows the results of the analysis for each predictor variable. It was not possible to fit a multiple model, with more than one variable being a predictor together with another, among the variables evaluated. For this reason, the univariate analysis is shown in Table 2, where it is observed that, in isolation, the variables NW and NH were configured as predictors of sex. For this reason, Table 2 describes only the univariate analysis.

In the data validation step ($n=157$), the accuracy of sex prediction was 52.86% for NW and 64.96% for NH, considering the test cutoff points indicated (Table 3).

Table 3 describes the values obtained for the sample used in the test phase of the new cutoff points, which were applied to the test sample, generating the values of sensitivity (Se), specificity (Sp), positive predictive value (PV+), negative predictive value (PV-), positive likelihood (LR+), negative likelihood (LR-), and accuracy (Acc).

For NW and NH, moderate accuracies were obtained. Positive and negative predictive values were moderate and high, respectively, indicating moderate and high probabilities of correct classification, for females and males, respectively.

Table 3 also shows the positive and negative predictive values (LR+ and LR-) of the NH and NW variables, that is the probability that subjects classified by the model as male truly are male, and the probability that subjects classified by the model as female truly are female.

Table 1. Distribution of measurements of OH, OW, NW, NH, OI, NI, and RONI, and comparison between males and females

Variable	Predict categories	Measurement value Mean (SD)	p-value
**OH**	Female	38.42 (±5.14)	0.129
	Male	39.44 (±5.32)	
**OW**	Female	41.54 (±4.01)	0.252
	Male	42.15 (±4.36)	
**NW**	Female	34.15 (±5.07)	0.030
	Male	35.66 (±5.70)	
**NH**	Female	49.14 (±7.36)	0.028
	Male	51.25 (±7.59)	
**OI**	Female	0.92 (±0.09)	0.363
	Male	0.93 (±0.10)	
**NI**	Female	0.70 (±0.09)	0.984
	Male	0.70 (±0.09)	
**RONI**	Female	1.33 (±0.20)	0.453
	Male	1.36 (±0.23)	

Legend: OH (orbital height); OW (orbital width); NW (nasal width); NH (nasal height); OI (orbital index = OH/OW); NI (nasal index = NW/NH); RONI (OI/NI). Student's t-test. Significance level = 5%.

Table 2. Results of sex prediction from OH, OW, NW, NH, OI, NI, and RONI

Variable	Sex	p-value	OR (CI 95%)
**OH**	Female	0.130	1
	Male		1.03 (0.98 - 1.08)
**OW**	Female	0.252	1
	Male		1.03 (1.02 - 1.10)
**NW**	Female	0.031	1
	Male		1.05 (1.01 - 1.08)
**NH**	Female	0.030	1
	Male		1.03 (1.01 - 1.05)
**OI**	Female	0.376	1
	Male		3.12 (0.61 - 5.62)
**NI**	Female	0.989	1
	Male		0.98 (-1.56 - 3.53)
**RONI**	Female	0.462	1
	Male		1.53 (0.39 – 2.66)

Legend: OH (orbital height); OW (orbital width); NW (nasal width); NH (nasal height); OI (orbital index = OH/OW); NI (nasal index = NW/NH); RONI (OI/NI). Logistic regression. OR=Odds ratio. IC 95% = Confidence interval 95%. Significance level = 5%.

Table 3. Contingency table and predictive values for classification of individuals based on NW and NH measurements

Variable		Real classification		Se	Sp	PV+	PV-	LR+	LR-	Acc
		Female	Male							
NW classification	Female	43	58	72.88% (65.93-79.83)	40.81% (33.12-48.50)	42.57% (34.84-50.30)	71.42% (64.35-78.49)	1.23 (0.54-1.92)	0.66 (0.58-0.73)	52.86% (45.05-60.67)
	Male	16	40							
NH classification	Female	33	29	55.93% (48.16-63.70)	70.40% (63.26-77.54)	53.22% (45.41-61.03)	72.63% (65.66-79.60)	1.88 (1.05-2.71)	0.62 (0.54-0.69)	64.96% (57.50-72.42)
	Male	26	69							

Legend: Se = Sensitivity; Sp = Specificity; PV+ = Positive predictive value; PV- = Negative predictive value; LR+ = Positive likelihood ratio; LR- = Negative likelihood ratio; Acc=Accuracy.

Discussion

Human beings are unique in terms of their physical growth and development, which means that related measurements can be used to identify individuals based on physical variability. The morphological features of the skull are useful in the identification of dead and living persons [16, 17]. Measurements of facial anatomy, including sinuses, nasal cavity, or orbital cavity, are now widely used in forensic identification, even if it is necessary to carefully differentiate between different ethnic groups. Face measurements are also made for various health purposes, such as in the field of dental prostheses, cosmetic surgery, orthodontics and face masks. In particular, face analysis and proportions have been shown to be important for facial plastic surgeons to evaluate the face during the planning stages of facial reconstructive and cosmetic surgery [18].

Craniometric parameters including NI and OI have been investigated in prior research to estimate sex in forensic medicine. Preliminary knowledge of these measurements is essential for correct application since they vary from one population to another. The literature shows that there are significant differences in orbital and nasal morphometry between individuals in relation to age, sex, and ethnicity [19].

This study was conducted to establish the orbital and nasal indexes of Kosovar adults using skull CT scans. Previous studies have shown that measurements for the dimensions of orbital and nasal cavities taken from human skulls are similar to those obtained by CT scans [20], and the

consistency of measurements using CT images has been evaluated in the last decade [21]. Our analysis was performed on head CT scans of patients with headache disorders, neurological deficits (e.g., poststroke), epilepsy, and other medical issues. This research excluded individuals with a history of bone pathology, and the sample can therefore be considered a “normal population” [22].

The human nose, as the most protruding part of the face, is variable in size and shape [23, 24]. Divergence of nose shape and prominence can be explained by ethnic influences and environmental and climatic conditions [23-25]. In the present study using CT scans, the measurements of both the nasal aperture and orbital cavity were higher in males compared to females. These observations are in agreement with the results of Vidya et al., who examined dry skulls of South Indian origin [25], and with those of Nasir et al., who studied nasal indexes across four Indian states [26]. Similarly, the nasal index, calculated from height and weight measurements of the nasal cavity, was found to be higher in males than in females in this research, in line with the findings of Staka et al. (on another Kosovar population), Hegazy (on Egyptians), Sforza et al., and Franciscus and Long [12, 13, 27, 28]. Our results also showed that Kosovar adult males have a mean orbital index of 0.93 (0.10), while the equivalent score for adult females is 0.92 (0.09) (Table 1). Results from this study concur with previous research that found a significant sex difference ($p<0.05$) in the orbital indexes of Indian and Nigerian adults [29]. The present study does not support other investigations that have noted a higher OI in females than in males. The differences seen in these populations may be attributed to the different age groups of subjects across various studies [30]. Also, in our study the mean orbital height and weight measurements for females and males are half of those recorded in Indian, Nigerian, Malawian, Bini, and Urhobo populations [31]. We found that there was a significant difference in the orbital index between males and females, mainly because males exhibit wider orbital cavities than females. Some authors have reported differences in the volume of OI between males and females, whereas others have shown no such difference [32-35].

Lusted and Keats reported that orbital parameters obtained from radiographs are slightly different from those obtained from direct measurement of human skulls and that this difference could be attributed to the magnification factor of X-ray machines [35]. Indeed, the results of the present study might have been affected by the magnification factor and therefore may not present completely true measurements of the orbital or nasal cavity. Other differences between our

findings and those of other authors could be due to different patterns of craniofacial growth caused by racial, ethnic, social, environmental, and nutritional factors [28, 36, 37].

Differences in nose shape and appearance are influenced by ethnic factors and environmental climatic conditions. A study on the Dayak Kenyah population in North Kalimantan, Indonesia, found that the predominant nasal shape was mesorrhine and attributed this to environmental factors, concluding that differences between nasal shapes may be used to trace geographic origins [38]. In a study on a South Indian population, using MDCT 2D scans to measure nasal index and nasal parameters, Kotian et al. found that the measurements of nasal aperture were greater in males compared to females [15]; these findings are in agreement with the present study. Regardless of sexual dimorphism, it is predictable that ethnic groups in the same climatic regions should have similarities in their nasal indexes. In any case, the point that is most relevant for forensic investigations is that anthropometric data of the face and nose would be useful for sex determination, and Svitlana and Themozhi have suggested that nasal proportions are indeed a useful anthropometrical tool in determination of sex in forensic science and classification of fossil remains [39].

This study indicates that NW and NH have potential to classify individuals as female and male with predictive accuracy of around 52.86% and 64.96%, respectively. Anthropological studies agree that variations in the nasal index are related to the climate. Variation in the bony nasal cavity probably stems from man’s ability to adapt to the environment. The peculiarity of this study is the fact that a different predictivity in the estimation of sex, with statistical significance, lies not in the calculation of the NI, but in the individual measurements which, if compared, form the NI (NW and NH). In particular, females tend to have a lower NW than that of males, as well as a higher NH. However, it should be noted that, as limitation, the study has moderate accuracy with not very high power to classify sex. In a larger pool of samples or in other Caucasian populations we could find even higher levels of accuracy.

To understand whether this nasal anatomical variation is due to the adaptation of the Kosovar population to the specific environment or whether it concerns other ethnic groups, broadly speaking, it would be useful to extend the analysis to other neighboring and non-neighboring populations.

Conclusion

Sex estimation is essential for completing an individual's biological profile and therefore constitutes an important part of the human identification process in the forensic field. It also has significant ethical and legal implications [40-42].

The current anthropometric study found that the metric parameters necessary for the calculation of NI showed pronounced sexual dimorphism in a large Kosovar population. Since the equation developed in the stepwise discriminant analysis was intended for this specific population, it may produce different results for other population groups. Further studies are recommended to evaluate and characterize orbital and nasal parameters among different populations; it could be useful test the discriminant function in populations from different countries, and study the dependence of anthropometric data on sex and age. Therefore, conducting a comparative study between different groups would be very interesting in the field of personal identification for forensic purposes. In the future, studies could be conducted to compare the measurements made on CT scans with those performed directly on the skulls and verify if there is an anthropometric variability that could make the results different.

Acknowledgments

Thanks to Jemma Dunnill for proofreading the manuscript.

References

1. Catalina-Herrera CJ. Morphometric study of the orbit's base in male and female skulls of Spaniards. Bull Assoc Anat (Nancy). 1988;72(217-218):5-7.
2. Giles E, Elliot O. Race identification from cranial measurements. J Forensc Sci. 1962; 7: 147-57.
3. Nitek S, Wysocki J, Reymond J, Piasecki K. Correlations between selected parameters of the human skull and orbit. Med Sci Monit. 2009;15(12):BR370-BR377.

4. Forbes G, Gehring DG, Gorman CA, Brennan MD, Jackson IT. Volume measurements of normal orbital structures by computed tomographic analysis. AJR Am J Roentgenol. 1985;145(1):149-154. doi:10.2214/ajr.145.1.149
5. Karampatakis V, Natsis K, Gigis P, Stangos NT. Orbital depth measurements of human skulls in relation to retrobulbar anesthesia. Eur J Ophthalmol. 1998;8(2):118-120.
6. Ezeuko VC, Om'Iniabohs FAE. Radiologic evaluation of the orbital index among the Igbo ethnic group of Nigeria. Eur J Anat. 2015; 19: 9-14.
7. Dilmen G, Köktener A, Turhan NO, Tez S. Growth of the fetal lens and orbit. Int J Gynaecol Obstet. 2002;76(3):267-271. doi:10.1016/s0020-7292(01)00581-1
8. Merz E, Bahlmann F, Weber G. Volume scanning in the evaluation of fetal malformations: a new dimension in prenatal diagnosis. Ultrasound Obstet Gynecol. 1995;5(4):222-227. doi:10.1046/j.1469-0705.1995.05040222.x
9. Rajangam S, Kulkarni RN, Quadrilos L, Sreenivasulu S. Orbital dimensions. Indian J Anat. 2012; 1: 5-9.
10. Mehta M, Saini V, Nath S, Patel NM, Menon SK. CT scan images to determine the origin from craniofacial indices for Gujarati population. Journal of Forensic Radiology and Imaging. 2014; 2: 64-71.
11. Oladipo GS, Olabiyi AO, Oremosu AA, NoronhaCC. Nasal indices among major ethnic groups in Southern Nigeria. SRE. 2007; 2(1): 20-22.
12. Staka G, Dragidella F, Disha M. Anthropometric study of nasal index of the Kosovo Albanian population. Online Journal of Anthropology. 2012; 8: 457-62.
13. Hegazy AA. Anthropometric study of nasal index of Egyptians. Int J Anat Res. 2014; 2: 761-7.
14. Ravichandran S, Yuvraj Babu K, Mohanraj KG. Correlation of facial and nasal index in gender determination. Drug Invention Today. 2018; 10(12).
15. Kotian R, Bakkannavar SM, Shekhar H, Pradhan P, Nayak VC. Sex determination based on nasal index and nasal parameters using (big bore 16 slice) multidetector computed tomography 2D scans. Indian J Forensic Community Med. 2015; 2:167-71.
16. Hsiao TH, Chang HP, Liu KM. Sex determination by discriminant function analysis of lateral radiographic cephalometry. J Forensic Sci. 1996;41(5):792-795.

17. Cameriere R, Zolotenkova GV, Kuznetsov IA, Scendoni R, Pigolkin YI. Sex estimation based on the anthropometric measurements of thyroid cartilage using discriminant analysis. Egypt J Forensic. 2021; Sci. 11.

18. Shahbazi Z, Ardalani H, Maleki M. Aesthetics of Numerical Proportions in Human Cosmetic Surgery. World J Plast Surg. 2019 ; 8(1):78-84.

19. Kanjani V, Rani A, Kanjani D. Morphometric analysis of the orbital aperture in north indian population: a retrospective digital forensic study. Int J Appl Basic Med Res. 2019;9(2):85-88. doi:10.4103/ijabmr.IJABMR_404_18

20. Patil KR, Mody RN. Determination of sex by discriminant function analysis and stature by regression analysis: a lateral cephalometric study. Forensic Sci Int. 2005;147(2-3):175-180. doi:10.1016/j.forsciint.2004.09.071

21. Krishan K. Estimation of stature from cephalo-facial anthropometry in north Indian population. Forensic Sci Int. 2008;181(1-3):52.e1-52.e526. doi:10.1016/j.forsciint.2008.08.0

22. Veyre-Goulet SA, Mercier C, Robin O, Guérin C. Recent human sexual dimorphism study using cephalometric plots on lateral teleradiography and discriminant function analysis. J Forensic Sci. 2008;53(4):786-789. doi:10.1111/j.1556-4029.2008.00759.x

23. Hsiao TH, Tsai SM, Chou ST, Pan JY, Tseng YC, Chang HP, et al. Sex determination using discriminant function analysis in children and adolescents: a lateral cephalometric study. Int J Legal Med. 2010;124(2):155-160. doi:10.1007/s00414-009-0412-1

24. Naikmasur VG, Shrivastava R, Mutalik S. Determination of sex in South Indians and immigrant Tibetans from cephalometric analysis and discriminant functions. Forensic Sci Int. 2010;197(1-3):122.e1-122.e1226. doi:10.1016/j.forsciint.2009.12.052

25. Vidya CS, Shamasundar NM, Manjunatha B, Raichurkar K. Evaluation of size and volume of maxillary sinus to determine sex by 3D computerized tomography scan method using dry skulls of South Indian origin. International Journal of Current Research and Review. 2013; 5: 97.

26. Nasir N, Asad MR, Muzammil K, Hassan A, Alshalwi T, Khan MR. Anthropometric study of nasal indices in four Indian Stat. Cli. Pract. 2021; 18: 1620-5.

27. Sforza C, Dellavia C, Colombo A, Serrao G, Ferrario VF. Nasal dimensions in normal subjects: conventional anthropometry versus computerized anthropometry. Am J Med Genet A. 2004; 130: 228-33. doi: 10.1002/ajmg.a.30275. PMID: 15378539.

28. Franciscus RG, Long JC. Variation in human nasal height and breadth. Am J Phys Anthropol. 1991;85(4):419-427. doi:10.1002/ajpa.1330850406
29. Oladipo GS, Eroje MA, Fahwehinmi HB. Anthropometric comparison of nasal indices between Andoni and Okrika tribes of Rivers State, Nigeria. International Journal of Medicine and Medical Sciences 2009; 1: 135-7.
30. Ahmed AA, Mohammed H, Mohamed H. Sex determination from cranial measurements in recent northern Sudanese. Khartoum Med J; 2011; 1: 539–47
31. O'Donnell C, Iino M, Mansharan K, Leditscke J, Woodford N. Contribution of postmortem multidetector CT scanning to identification of the deceased in a mass disaster: Experience gained from the 2009 Victorian bushfires. Forensic Sci Int. 2011;205(1-3):15-28. doi:10.1016/j.forsciint.2010.05.026.
32. Amin MF, Hassan EI. Sex identification in Egyptian population using Multidetector Computed Tomography of the maxillary sinus. J Forensic Leg Med. 2012;19(2):65-69. doi:10.1016/j.jflm.2011.10.005
33. Sukumar S, Yadav S, Vipinkumar G. Sex determination by mastiod process in South Indian population by 3D-computer tomography imaging. Int J PharmBiol Sci. 2012; 4:193–5.
34. de Froidmont S, Grabherr S, Vaucher P, De Cesare M, Egger C, Papageorgopoulou C, et al. Virtual anthropology: a comparison between the performance of conventional X-ray and MDCT in investigating the trabecular structure of long bones. Forensic Sci Int. 2013;225(1-3):53-59. doi:10.1016/j.forsciint.2012.10.029
35. Lusted LB, Keats ET. Geometric distortion of roentgen image and its correction. In: Keats TE, editor. Atlas of roentgenographic measurements, Chicago Year Book Medical Pub; 1977. pp. 1-4.
36. Lusted LB, Keats ET. Geometric distortion of roentgen image and its correction. In: Keats TE, editor. Atlas of roentgenographic measurements, Chicago Year Book Medical Pub; 1977. p. 30
37. Howale D, Pradhan R, Jain L, Lekharu R. The calculation of various craniofacial indices in Maharastra population. Int J Curr Res; 2012; 4: 162–6.
38. Marini MI, Angrosidy H, Kurniawan A, Margareth MS. The anthropological analysis of the nasal morphology of Dayak Kenyah population in Indonesia as a basic data for forensic identification. Translational Research in Anatomy. 2020; 19.

39. Svitlana AS, Themozhi MS. Study of nasal index-a review. IJARIIE. 2019; 5: 110-5.
40. De Micco F, Martino F, Campobasso CP. Ethical issues in age assessment by the third molar development. Australian Journal of Forensic Sciences. 2022; 54:88-99.
41. Angelakopoulos N, Galić I, De Luca S, Campobasso CP, Martino F, De Micco F, et al.. Skeletal age assessment by measuring planar projections of carpals and distal epiphyses of ulna and radius bones in a sample of South African subadults, Australian Journal of Forensic Sciences, 2022; 54:1.DOI: 10.1080/00450618.2020.1766111
42. Bakkannavar SM., Manjunath S, Nayak VC, Pradeep Kumar G. Canine index – A tool for sex determination, Egyptian Journal of Forensic Sciences, Volume 5, Issue 4, 2015, 157-161

Figure 1

Click here to access/download;Figure;Fig. 1.tif

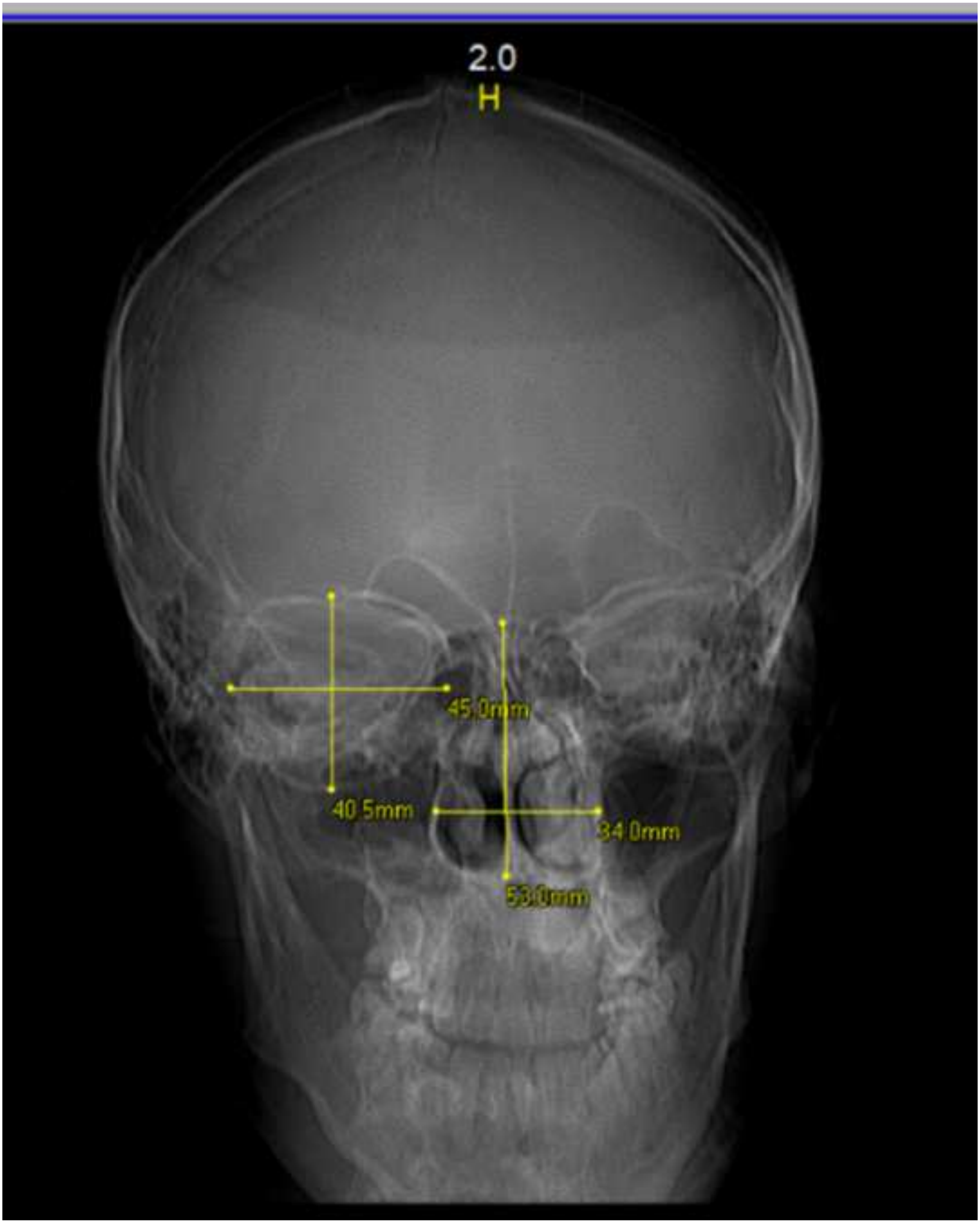

Figure 2

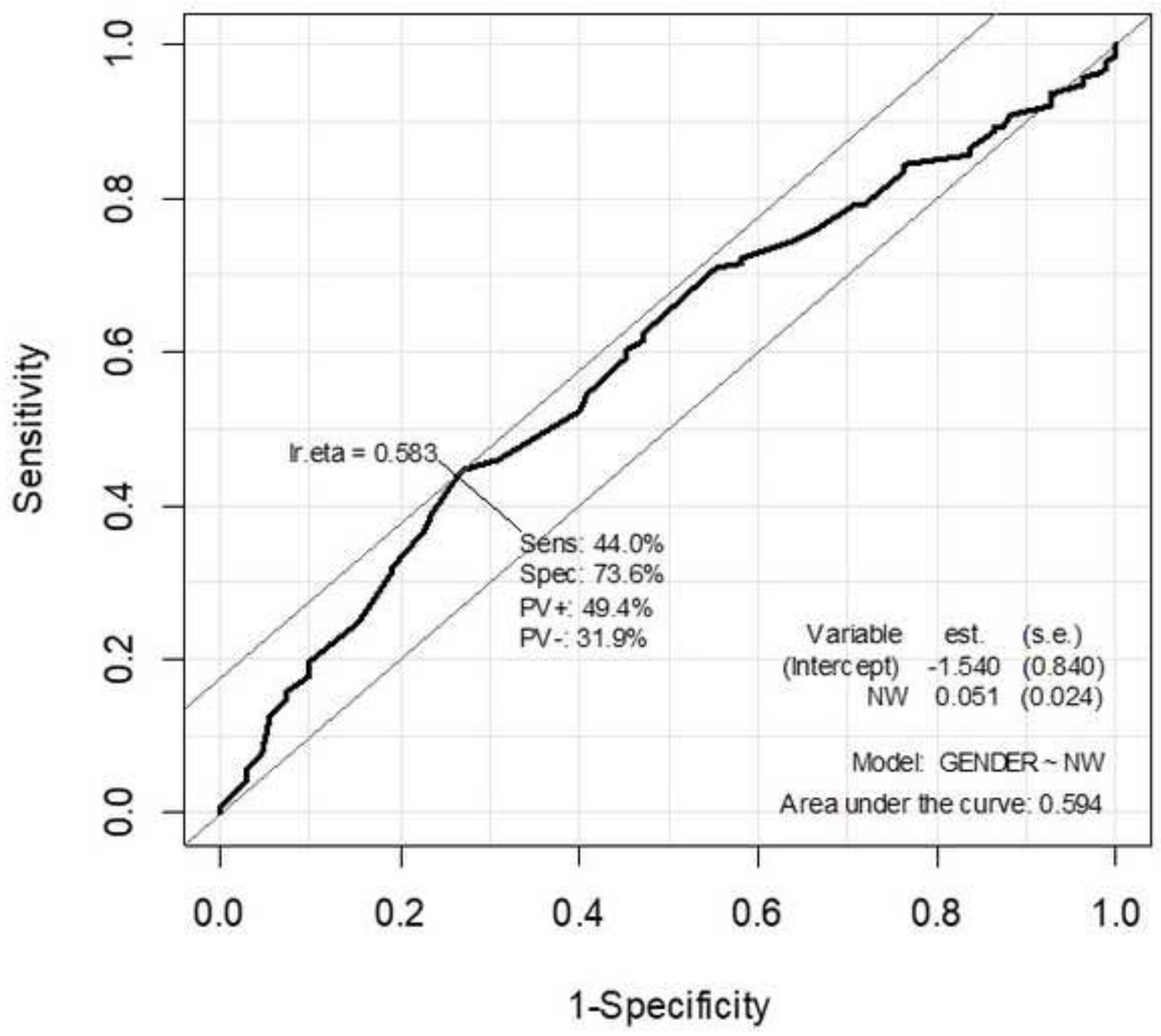

lr.eta = 0.583
Sens: 44.0%
Spec: 73.6%
PV+: 49.4%
PV-: 31.9%
Variable est. (s.e.)
(Intercept) -1.540 (0.840)
NW 0.051 (0.024)
Model: GENDER ~ NW
Area under the curve: 0.594
Sensitivity
0.0
0.2
0.4
0.6
0.8
1.0
1-Specificity

Figure 3

Click here to access/download;Figure;Fig. 3.tif

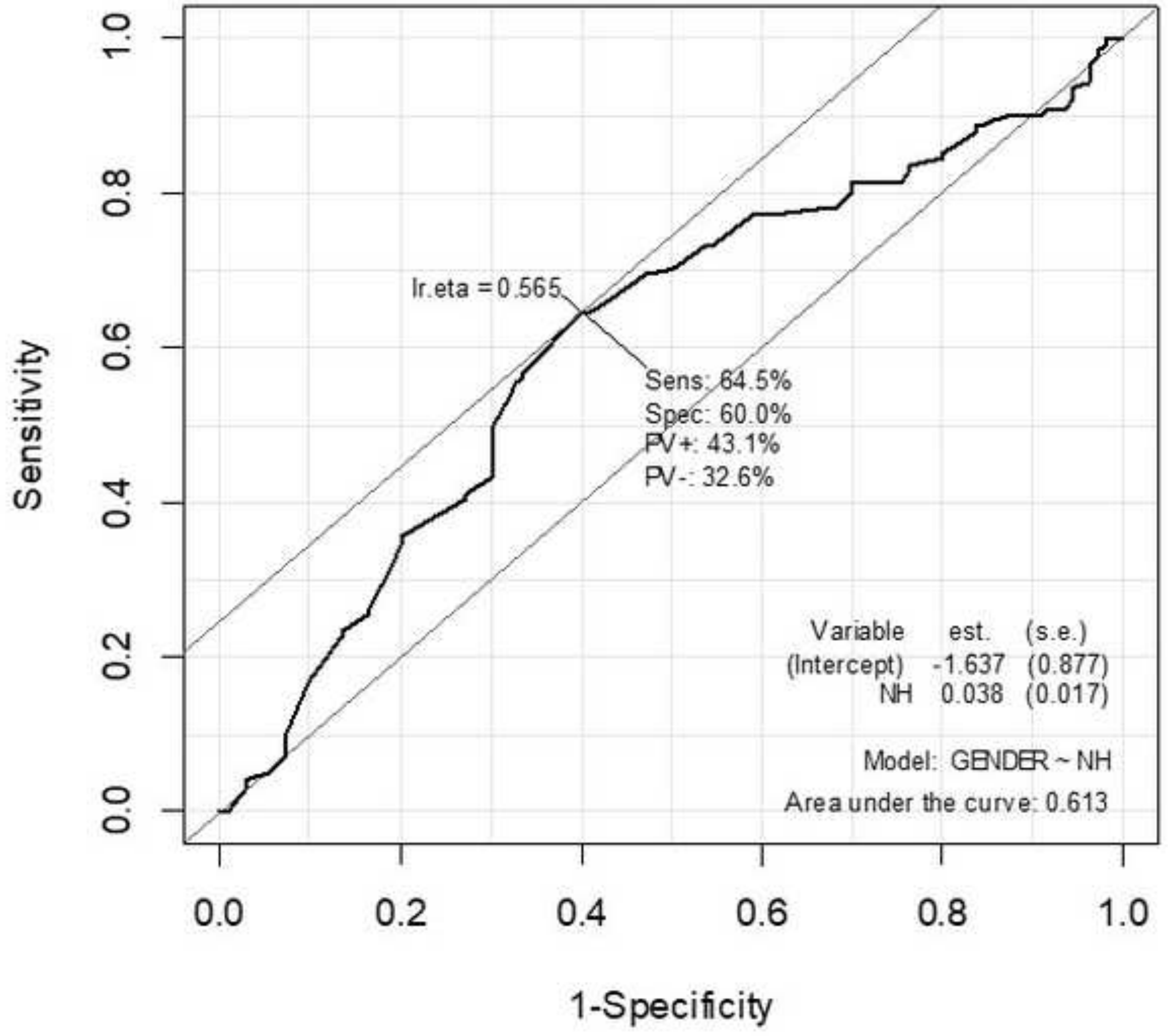

Anthropometric analysis of orbital and nasal parameters for sexual dimorphism: new anatomical evidences in the field of personal identification

New anatomical evidences for forensic personal identification

Roberto Scendoni [1,], Jeta Kelmendi [2], Isabella Lima Arrais Ribeiro [3], Mariano Cingolani [1], Francesco De Micco [4 *], Roberto Cameriere [5]

[1] Department of Law, Institute of Legal Medicine (AgEstimation Project), University of Macerata, Macerata, Italy
[2] Faculty of Medicine, Department of Orthodontic, University of Prishtina, Kosovo. Alma Mater Europaea, Campus Rezonanca, Prishtina, Kosovo
[3] Postgraduate Program in Dentistry, Federal University of Paraíba, Campus I, João Pessoa, PB, 58051900, Brazil
[4] Bioethics and Humanities Research Unit, Campus Bio-Medico University of Rome, 00128 Rome, Italy
[5] Department of Forensic Medicine (AgEstimation Project), University of Molise, Italy

*Corresponding author
E-mail: f.demicco@policlinicocampus.it (FDM)

Abstract

Orbital and nasal parameters among modern humans show considerable variation, which affects facial shape, and these characteristics vary according to race, region, and period in evolution. The aim of the study was to ascertain whether there are sex differences in the orbital and/or nasal indexes (OI and NI) and/or the single measurements used to calculate these in a Kosovar population. The following parameters were taken into consideration: orbital height (OH), orbital width (OW), nasal height (NH), and nasal width (NW). The ratios between orbital index/nasal index (RONI) were calculated. All measurements were obtained from a population sample comprising 408 individuals. The sample was randomly divided into two subsamples, one constituting approximately 2/3 (n=251) of the initial sample, and the other containing approximately 1/3 of the initial sample (n=157). This division was important for the steps of training (2/3 of sample) and testing (validation) (1/3 of sample) variables of interest for the prediction of sex. Therefore, 251 individuals were evaluated, 110 females and 141 males, with a mean age of 37.41 (±20.56) and 33.87 (±19.58), respectively. The data were collected from head CT scans using digital calipers and statistically analyzed (Kolmogorov-Smirnov normality test, Levene's test, Student's t-test and logistic regression). After prediction analysis, the cutoff points were selected: 36.76 for NW measurements, and 50.00 for NH measurements. For two variables, measurements below these cutoff values indicated female sex and measurements above indicated male sex. The accuracy in sex prediction was 52.86% (CI95% = 45.05% - 60.67%) for NW and 64.96% for NH (CI95% = 57.50% – 72.42%). The difference between male and female indexes was statistically significant ($P < 0.05$). The anthropometric study revealed that only NW and NH are configured as predictors of sexual dimorphism. It could be useful to increase the number of samples to test the discriminant function in other population groups.

Introduction

Assessing ethnicity and identifying skeletal remains are important and difficult tasks for forensic scientists. Numerous methods using craniofacial measurements and indexes are available and useful for these purposes. The manner of determining the parameters needed for the estimation of these directories depends on the sort of samples used [1]. Extensive research has been conducted

worldwide on the dimensions and volumes of the orbits and nasal cavity. Calipers are still commonly used as linear measuring instruments to measure the dimensions of the orbital and nasal margins [2, 3]. However, advanced radiological techniques such as computed tomography (CT) scanners have proven to be a better choice since they facilitate sample collection [2]. The orbital index (OI) and nasal index (NI) are calculated using measurements between several landmarks in the respective facial cavity [3]. Orbital and nasal cavity dimensions and volumes, in various populations, have been investigated by many authors [4-6], and different populations have been found to exhibit considerable variation in these values [7-10]. Indeed, population-based variations are predictable results of evolutionary processes; natural selection acts on inheritable mutations, which may relate to current environmental pressures, genetic implication, past and present hybridization between geographically distinct populations, and the present selective adaptation of human varieties to their environment.

Overall, these studies have indicated some level of ethnic and racial variation in the OI and NI of various population groups. In modern human groups, the appearance of the orbital and nasal cavities varies considerably [5]. Craniofacial morphometric analysis is conducted using OI and NI parameters, based on the ratio of the orbital/nasal height to its width multiplied by 100. These ratios affect the shape of the face and vary with race, regions within the same race, and periods in evolution [11-14].

However, to date there has been no morphometric study on OI and NI and their relationship to biological sex prediction in the Kosovar population. Therefore, this study of the orbital and nasal morphometry in the skulls of a Kosovar population using a CT scanner represents an important contribution to the literature which will expand knowledge in various fields, such as forensic medicine, and help to explore trends in evolutionary and ethnic differences, among different races in particular, where forensic data is not available. Thus, the main objective of this research is to ascertain whether there are sex differences in the orbital and/or nasal indexes and/or the single measures used to calculate these.

Materials and Methods

A retrospective study design was used in this research involving the Kosovar ethnic group living in Kosovo. Inclusion criteria were Kosovar males and females over the age of 18, who underwent

CT scan to investigate neurological symptoms. Exclusion criteria included growth diseases, endocrine disorders or osteodystrophy, previous fractures of the of the skull and the facial massif. Finally, all blurred TC images were disregarded. The CT scans were anonymously extracted and limited data were extracted that were strictly necessary and relevant for the conduct of the study. The extracted data are all available in the manuscript. A sample of 408 individuals was randomly divided into two subsamples, one constituting approximately 2/3 (n=251) of the initial sample, and the other containing approximately 1/3 of the initial sample (n=157). This division was important for the steps of training (2/3 of sample) and testing (validation) (1/3 of sample) variables of interest for the prediction of sex. Therefore, 251 individuals were evaluated, 110 females and 141 males, with a mean age of 37.41 (±20.56) and 33.87 (±19.58), respectively. The sample of the present study was calculated based on the effects observed in the study by Kotian et al. [15]. From this study, the mean effect size of difference between groups of 0.20 (Cohen's d) was observed. Adopting a 5% type I error (95% study confidence level), a 20% type II error (80% study power), the effect size observed in the mentioned study and, considering the comparison tests of the groups of the bilateral type (two-sided), it was calculated that at least 394 sample units were necessary for the accomplishment of the study. As they are available, we conducted the study with 408 sample units. The calculations were performed using the G*Power statistical software.

The study was carried out on frontal views of head CT scans performed on patients who had attended radiology units (private and public) in various cities in Kosovo. The height and width of the orbital cavity and nasal aperture were measured on the CT scan of the frontal bone using a digital ruler (in mm). The following parameters were measured: orbital height (OH) and nasal height (NH) – the maximum distance between the upper and lower margins of the orbital cavity rim and nasal aperture rim (Fig 1); orbital width (OW) and nasal width (NW) – the distance from the midpoint of the medial margin of the orbital/nasal aperture to the midpoint of the lateral margin of the same orbit/nasal aperture. The mean orbital and nasal indexes were calculated by the formula: height/width x 100. Only normal CT scans were used (cases with bone pathology and fractures were excluded from the study). Finally, the ratios between orbital index/nasal index (RONI) were calculated.

Fig. 1. Plain film showing the orbital and nasal margins and measurements of orbital/nasal width and height.

The data were analyzed in R software (version 3.6.3, R Core Team, R Foundation for Statistical Computing, Vienna, Austria). Initially, the data were tested for normal distribution, verifying a normal distribution for all the quantitative variables (p>0.05; Kolmogorov-Smirnov normality test). Sequentially, the values of the variables OH, OW, NW, NH, OI, NI, and RONI were compared between male and female subjects (Levene's test and Student's t-test), and dependence of outcome (sex prediction) on each variable of interest was evaluated by logistic regression, using female sex as the reference group. The female gender was chosen as the reference category because the measures of interest were lower for females and leaving this category as a reference makes the interpretation of the odds ratio findings more understandable. Receiver operating characteristic curves (ROCs) (Figs 2 and 3) were generated for the NW and NH variables, these being the most accurate predictors of sex. The best cutoff points for each variable of interest were obtained from the equation "Ir.eta=1/1+e$^{-(\alpha + \beta X)}$": 36.76 for NW measurements, and 50.00 for NH measurements. For the two variables under study, values below the cutoff score were interpreted as indicating female sex, while values above the cutoff score were taken to indicate male sex. In the second phase of this study, these cutoff points were tested in an independent sample of 157 other individuals. A contingency table (confusion matrix) was generated, along with the respective predictive values of sensitivity and specificity (Se and Sp), positive and negative predictive values (PV+ and PV-), the positive and negative likelihood ratios (LR+ and LR-), and accuracy (Acc), including their respective confidence intervals 95% (CI95%). The significance level was 5% for all analyses. The Ethical Issues Committee of the Kosovo Chamber of Dentists approved the study (nr. 27, 18/05/2022).

Fig 2. Receiver operating characteristic curve for prediction analysis of sex from NW

Fig 3. Receiver operating characteristic curve for prediction analysis of sex from NH.

Results

251 individuals were evaluated, 110 females and 141 males, with a mean age of 37.41 (±20.56) and 33.87 (±19.58), respectively, there was no statistically significant difference in age between the sexes. (p=0.232; Student's t-test).

Table 1 presents the distributions and comparisons of measurements for males and females.

Table 2 shows the results of the analysis for each predictor variable. It was not possible to fit a multiple model, with more than one variable being a predictor together with another, among the variables evaluated. For this reason, the univariate analysis is shown in Table 2, where it is observed that, in isolation, the variables NW and NH were configured as predictors of sex. For this reason, Table 2 describes only the univariate analysis.

In the data validation step (n=157), the accuracy of sex prediction was 52.86% for NW and 64.96% for NH, considering the test cutoff points indicated (Table 3).

Table 3 describes the values obtained for the sample used in the test phase of the new cutoff points, which were applied to the test sample, generating the values of sensitivity (Se), specificity (Sp), positive predictive value (PV+), negative predictive value (PV-), positive likelihood (LR+), negative likelihood (LR-), and accuracy (Acc).

For NW and NH, moderate accuracies were obtained. Positive and negative predictive values were moderate and high, respectively, indicating moderate and high probabilities of correct classification, for females and males, respectively.

Table 3 also shows the positive and negative predictive values (LR+ and LR-) of the NH and NW variables, that is the probability that subjects classified by the model as male truly are male, and the probability that subjects classified by the model as female truly are female.

Table 1. Distribution of measurements of OH, OW, NW, NH, OI, NI, and RONI, and comparison between males and females

Variable	Predict categories	Measurement value Mean (SD)	p-value
**OH**	Female	38.42 (±5.14)	0.129
	Male	39.44 (±5.32)	
**OW**	Female	41.54 (±4.01)	0.252
	Male	42.15 (±4.36)	
**NW**	Female	34.15 (±5.07)	0.030
	Male	35.66 (±5.70)	
**NH**	Female	49.14 (±7.36)	0.028
	Male	51.25 (±7.59)	
**OI**	Female	0.92 (±0.09)	0.363
	Male	0.93 (±0.10)	
**NI**	Female	0.70 (±0.09)	0.984
	Male	0.70 (±0.09)	
**RONI**	Female	1.33 (±0.20)	0.453
	Male	1.36 (±0.23)	

Legend: OH (orbital height); OW (orbital width); NW (nasal width); NH (nasal height); OI (orbital index = OH/OW); NI (nasal index = NW/NH); RONI (OI/NI). Student's t-test. Significance level = 5%.

Table 2. Results of sex prediction from OH, OW, NW, NH, OI, NI, and RONI

Variable	Sex	p-value	OR (CI 95%)
**OH**	Female	0.130	1
	Male		1.03 (0.98 - 1.08)
**OW**	Female	0.252	1
	Male		1.03 (1.02 - 1.10)
**NW**	Female	0.031	1
	Male		1.05 (1.01 - 1.08)
**NH**	Female	0.030	1
	Male		1.03 (1.01 - 1.05)
**OI**	Female	0.376	1
	Male		3.12 (0.61 - 5.62)
**NI**	Female	0.989	1
	Male		0.98 (-1.56 - 3.53)
**RONI**	Female	0.462	1
	Male		1.53 (0.39 – 2.66)

Legend: OH (orbital height); OW (orbital width); NW (nasal width); NH (nasal height); OI (orbital index = OH/OW); NI (nasal index = NW/NH); RONI (OI/NI). Logistic regression. OR=Odds ratio. IC 95% = Confidence interval 95%. Significance level = 5%.

Table 3. Contingency table and predictive values for classification of individuals based on NW and NH measurements

Variable		Real classification		Se	Sp	PV+	PV-	LR+	LR-	Acc
		Female	Male							
NW classification	Female	43	58	72.88%	40.81%	42.57%	71.42%	1.23	0.66	52.86%
	Male	16	40	(65.93-79.83)	(33.12-48.50)	(34.84-50.30)	(64.35-78.49)	(0.54-1.92)	(0.58-0.73)	(45.05-60.67)
NH classification	Female	33	29	55.93%	70.40%	53.22%	72.63%	1.88	0.62	64.96%
	Male	26	69	(48.16-63.70)	(63.26-77.54)	(45.41-61.03)	(65.66-79.60)	(1.05-2.71)	(0.54-0.69)	(57.50-72.42)

Legend: Se = Sensitivity; Sp = Specificity; PV+ = Positive predictive value; PV- = Negative predictive value; LR+ = Positive likelihood ratio; LR- = Negative likelihood ratio; Acc=Accuracy.

Discussion

Human beings are unique in terms of their physical growth and development, which means that related measurements can be used to identify individuals based on physical variability. The morphological features of the skull are useful in the identification of dead and living persons [16, 17]. Measurements of facial anatomy, including sinuses, nasal cavity, or orbital cavity, are now widely used in forensic identification, even if it is necessary to carefully differentiate between different ethnic groups. Face measurements are also made for various health purposes, such as in the field of dental prostheses, cosmetic surgery, orthodontics and face masks. In particular, face analysis and proportions have been shown to be important for facial plastic surgeons to evaluate the face during the planning stages of facial reconstructive and cosmetic surgery [18].

Craniometric parameters including NI and OI have been investigated in prior research to estimate sex in forensic medicine. Preliminary knowledge of these measurements is essential for correct application since they vary from one population to another. The literature shows that there are significant differences in orbital and nasal morphometry between individuals in relation to age, sex, and ethnicity [19].

This study was conducted to establish the orbital and nasal indexes of Kosovar adults using skull CT scans. Previous studies have shown that measurements for the dimensions of orbital and nasal cavities taken from human skulls are similar to those obtained by CT scans [20], and the

consistency of measurements using CT images has been evaluated in the last decade [21]. Our analysis was performed on head CT scans of patients with headache disorders, neurological deficits (e.g., poststroke), epilepsy, and other medical issues. This research excluded individuals with a history of bone pathology, and the sample can therefore be considered a "normal population" [22].

The human nose, as the most protruding part of the face, is variable in size and shape [23, 24]. Divergence of nose shape and prominence can be explained by ethnic influences and environmental and climatic conditions [23-25]. In the present study using CT scans, the measurements of both the nasal aperture and orbital cavity were higher in males compared to females. These observations are in agreement with the results of Vidya et al., who examined dry skulls of South Indian origin [25], and with those of Nasir et al., who studied nasal indexes across four Indian states [26]. Similarly, the nasal index, calculated from height and weight measurements of the nasal cavity, was found to be higher in males than in females in this research, in line with the findings of Staka et al. (on another Kosovar population), Hegazy (on Egyptians), Sforza et al., and Franciscus and Long [12, 13, 27, 28]. Our results also showed that Kosovar adult males have a mean orbital index of 0.93 (0.10), while the equivalent score for adult females is 0.92 (0.09) (Table 1). Results from this study concur with previous research that found a significant sex difference ($p<0.05$) in the orbital indexes of Indian and Nigerian adults [29]. The present study does not support other investigations that have noted a higher OI in females than in males. The differences seen in these populations may be attributed to the different age groups of subjects across various studies [30]. Also, in our study the mean orbital height and weight measurements for females and males are half of those recorded in Indian, Nigerian, Malawian, Bini, and Urhobo populations [31]. We found that there was a significant difference in the orbital index between males and females, mainly because males exhibit wider orbital cavities than females. Some authors have reported differences in the volume of OI between males and females, whereas others have shown no such difference [32-35].

Lusted and Keats reported that orbital parameters obtained from radiographs are slightly different from those obtained from direct measurement of human skulls and that this difference could be attributed to the magnification factor of X-ray machines [35]. Indeed, the results of the present study might have been affected by the magnification factor and therefore may not present completely true measurements of the orbital or nasal cavity. Other differences between our

findings and those of other authors could be due to different patterns of craniofacial growth caused by racial, ethnic, social, environmental, and nutritional factors [28, 36, 37].

Differences in nose shape and appearance are influenced by ethnic factors and environmental climatic conditions. A study on the Dayak Kenyah population in North Kalimantan, Indonesia, found that the predominant nasal shape was mesorrhine and attributed this to environmental factors, concluding that differences between nasal shapes may be used to trace geographic origins [38]. In a study on a South Indian population, using MDCT 2D scans to measure nasal index and nasal parameters, Kotian et al. found that the measurements of nasal aperture were greater in males compared to females [15]; these findings are in agreement with the present study. Regardless of sexual dimorphism, it is predictable that ethnic groups in the same climatic regions should have similarities in their nasal indexes. In any case, the point that is most relevant for forensic investigations is that anthropometric data of the face and nose would be useful for sex determination, and Svitlana and Themozhi have suggested that nasal proportions are indeed a useful anthropometrical tool in determination of sex in forensic science and classification of fossil remains [39].

This study indicates that NW and NH have potential to classify individuals as female and male with predictive accuracy of around 52.86% and 64.96%, respectively. Anthropological studies agree that variations in the nasal index are related to the climate. Variation in the bony nasal cavity probably stems from man’s ability to adapt to the environment. The peculiarity of this study is the fact that a different predictivity in the estimation of sex, with statistical significance, lies not in the calculation of the NI, but in the individual measurements which, if compared, form the NI (NW and NH). In particular, females tend to have a lower NW than that of males, as well as a higher NH. However, it should be noted that, as limitation, the study has moderate accuracy with not very high power to classify sex. In a larger pool of samples or in other Caucasian populations we could find even higher levels of accuracy.

To understand whether this nasal anatomical variation is due to the adaptation of the Kosovar population to the specific environment or whether it concerns other ethnic groups, broadly speaking, it would be useful to extend the analysis to other neighboring and non-neighboring populations.

Conclusion

Sex estimation is essential for completing an individual's biological profile and therefore constitutes an important part of the human identification process in the forensic field. It also has significant ethical and legal implications [40-42].

The current anthropometric study found that the metric parameters necessary for the calculation of NI showed pronounced sexual dimorphism in a large Kosovar population. Since the equation developed in the stepwise discriminant analysis was intended for this specific population, it may produce different results for other population groups. Further studies are recommended to evaluate and characterize orbital and nasal parameters among different populations; it could be useful test the discriminant function in populations from different countries, and study the dependence of anthropometric data on sex and age. Therefore, conducting a comparative study between different groups would be very interesting in the field of personal identification for forensic purposes. In the future, studies could be conducted to compare the measurements made on CT scans with those performed directly on the skulls and verify if there is an anthropometric variability that could make the results different.

Acknowledgments

Thanks to Jemma Dunnill for proofreading the manuscript.

References

1. Catalina-Herrera CJ. Morphometric study of the orbit's base in male and female skulls of Spaniards. Bull Assoc Anat (Nancy). 1988;72(217-218):5-7.
2. Giles E, Elliot O. Race identification from cranial measurements. J Forensc Sci. 1962; 7: 147-57.
3. Nitek S, Wysocki J, Reymond J, Piasecki K. Correlations between selected parameters of the human skull and orbit. Med Sci Monit. 2009;15(12):BR370-BR377.

4. Forbes G, Gehring DG, Gorman CA, Brennan MD, Jackson IT. Volume measurements of normal orbital structures by computed tomographic analysis. AJR Am J Roentgenol. 1985;145(1):149-154. doi:10.2214/ajr.145.1.149
5. Karampatakis V, Natsis K, Gigis P, Stangos NT. Orbital depth measurements of human skulls in relation to retrobulbar anesthesia. Eur J Ophthalmol. 1998;8(2):118-120.
6. Ezeuko VC, Om'Iniabohs FAE. Radiologic evaluation of the orbital index among the Igbo ethnic group of Nigeria. Eur J Anat. 2015; 19: 9-14.
7. Dilmen G, Köktener A, Turhan NO, Tez S. Growth of the fetal lens and orbit. Int J Gynaecol Obstet. 2002;76(3):267-271. doi:10.1016/s0020-7292(01)00581-1
8. Merz E, Bahlmann F, Weber G. Volume scanning in the evaluation of fetal malformations: a new dimension in prenatal diagnosis. Ultrasound Obstet Gynecol. 1995;5(4):222-227. doi:10.1046/j.1469-0705.1995.05040222.x
9. Rajangam S, Kulkarni RN, Quadrilos L, Sreenivasulu S. Orbital dimensions. Indian J Anat. 2012; 1: 5-9.
10. Mehta M, Saini V, Nath S, Patel NM, Menon SK. CT scan images to determine the origin from craniofacial indices for Gujarati population. Journal of Forensic Radiology and Imaging. 2014; 2: 64-71.
11. Oladipo GS, Olabiyi AO, Oremosu AA, NoronhaCC. Nasal indices among major ethnic groups in Southern Nigeria. SRE. 2007; 2(1): 20-22.
12. Staka G, Dragidella F, Disha M. Anthropometric study of nasal index of the Kosovo Albanian population. Online Journal of Anthropology. 2012; 8: 457-62.
13. Hegazy AA. Anthropometric study of nasal index of Egyptians. Int J Anat Res. 2014; 2: 761-7.
14. Ravichandran S, Yuvraj Babu K, Mohanraj KG. Correlation of facial and nasal index in gender determination. Drug Invention Today. 2018; 10(12).
15. Kotian R, Bakkannavar SM, Shekhar H, Pradhan P, Nayak VC. Sex determination based on nasal index and nasal parameters using (big bore 16 slice) multidetector computed tomography 2D scans. Indian J Forensic Community Med. 2015; 2:167-71.
16. Hsiao TH, Chang HP, Liu KM. Sex determination by discriminant function analysis of lateral radiographic cephalometry. J Forensic Sci. 1996;41(5):792-795.

17. Cameriere R, Zolotenkova GV, Kuznetsov IA, Scendoni R, Pigolkin YI. Sex estimation based on the anthropometric measurements of thyroid cartilage using discriminant analysis. Egypt J Forensic. 2021; Sci. 11.
18. Shahbazi Z, Ardalani H, Maleki M. Aesthetics of Numerical Proportions in Human Cosmetic Surgery. World J Plast Surg. 2019 ; 8(1):78-84.
19. Kanjani V, Rani A, Kanjani D. Morphometric analysis of the orbital aperture in north indian population: a retrospective digital forensic study. Int J Appl Basic Med Res. 2019;9(2):85-88. doi:10.4103/ijabmr.IJABMR_404_18
20. Patil KR, Mody RN. Determination of sex by discriminant function analysis and stature by regression analysis: a lateral cephalometric study. Forensic Sci Int. 2005;147(2-3):175-180. doi:10.1016/j.forsciint.2004.09.071
21. Krishan K. Estimation of stature from cephalo-facial anthropometry in north Indian population. Forensic Sci Int. 2008;181(1-3):52.e1-52.e526. doi:10.1016/j.forsciint.2008.08.0
22. Veyre-Goulet SA, Mercier C, Robin O, Guérin C. Recent human sexual dimorphism study using cephalometric plots on lateral teleradiography and discriminant function analysis. J Forensic Sci. 2008;53(4):786-789. doi:10.1111/j.1556-4029.2008.00759.x
23. Hsiao TH, Tsai SM, Chou ST, Pan JY, Tseng YC, Chang HP, et al. Sex determination using discriminant function analysis in children and adolescents: a lateral cephalometric study. Int J Legal Med. 2010;124(2):155-160. doi:10.1007/s00414-009-0412-1
24. Naikmasur VG, Shrivastava R, Mutalik S. Determination of sex in South Indians and immigrant Tibetans from cephalometric analysis and discriminant functions. Forensic Sci Int. 2010;197(1-3):122.e1-122.e1226. doi:10.1016/j.forsciint.2009.12.052
25. Vidya CS, Shamasundar NM, Manjunatha B, Raichurkar K. Evaluation of size and volume of maxillary sinus to determine sex by 3D computerized tomography scan method using dry skulls of South Indian origin. International Journal of Current Research and Review. 2013; 5: 97.
26. Nasir N, Asad MR, Muzammil K, Hassan A, Alshalwi T, Khan MR. Anthropometric study of nasal indices in four Indian Stat. Cli. Pract. 2021; 18: 1620-5.
27. Sforza C, Dellavia C, Colombo A, Serrao G, Ferrario VF. Nasal dimensions in normal subjects: conventional anthropometry versus computerized anthropometry. Am J Med Genet A. 2004; 130: 228-33. doi: 10.1002/ajmg.a.30275. PMID: 15378539.

28. Franciscus RG, Long JC. Variation in human nasal height and breadth. Am J Phys Anthropol. 1991;85(4):419-427. doi:10.1002/ajpa.1330850406
29. Oladipo GS, Eroje MA, Fahwehinmi HB. Anthropometric comparison of nasal indices between Andoni and Okrika tribes of Rivers State, Nigeria. International Journal of Medicine and Medical Sciences 2009; 1: 135-7.
30. Ahmed AA, Mohammed H, Mohamed H. Sex determination from cranial measurements in recent northern Sudanese. Khartoum Med J; 2011; 1: 539–47
31. O'Donnell C, Iino M, Mansharan K, Leditscke J, Woodford N. Contribution of postmortem multidetector CT scanning to identification of the deceased in a mass disaster: Experience gained from the 2009 Victorian bushfires. Forensic Sci Int. 2011;205(1-3):15-28. doi:10.1016/j.forsciint.2010.05.026.
32. Amin MF, Hassan EI. Sex identification in Egyptian population using Multidetector Computed Tomography of the maxillary sinus. J Forensic Leg Med. 2012;19(2):65-69. doi:10.1016/j.jflm.2011.10.005
33. Sukumar S, Yadav S, Vipinkumar G. Sex determination by mastiod process in South Indian population by 3D-computer tomography imaging. Int J PharmBiol Sci. 2012; 4:193–5.
34. de Froidmont S, Grabherr S, Vaucher P, De Cesare M, Egger C, Papageorgopoulou C, et al. Virtual anthropology: a comparison between the performance of conventional X-ray and MDCT in investigating the trabecular structure of long bones. Forensic Sci Int. 2013;225(1-3):53-59. doi:10.1016/j.forsciint.2012.10.029
35. Lusted LB, Keats ET. Geometric distortion of roentgen image and its correction. In: Keats TE, editor. Atlas of roentgenographic measurements, Chicago Year Book Medical Pub; 1977. pp. 1-4.
36. Lusted LB, Keats ET. Geometric distortion of roentgen image and its correction. In: Keats TE, editor. Atlas of roentgenographic measurements, Chicago Year Book Medical Pub; 1977. p. 30
37. Howale D, Pradhan R, Jain L, Lekharu R. The calculation of various craniofacial indices in Maharastra population. Int J Curr Res; 2012; 4: 162–6.
38. Marini MI, Angrosidy H, Kurniawan A, Margareth MS. The anthropological analysis of the nasal morphology of Dayak Kenyah population in Indonesia as a basic data for forensic identification. Translational Research in Anatomy. 2020; 19.

39. Svitlana AS, Themozhi MS. Study of nasal index-a review. IJARIIE. 2019; 5: 110-5.
40. De Micco F, Martino F, Campobasso CP. Ethical issues in age assessment by the third molar development. Australian Journal of Forensic Sciences. 2022; 54:88-99.
41. Angelakopoulos N, Galić I, De Luca S, Campobasso CP, Martino F, De Micco F, et al.. Skeletal age assessment by measuring planar projections of carpals and distal epiphyses of ulna and radius bones in a sample of South African subadults, Australian Journal of Forensic Sciences, 2022; 54:1.DOI: 10.1080/00450618.2020.1766111
42. Bakkannavar SM., Manjunath S, Nayak VC, Pradeep Kumar G. Canine index – A tool for sex determination, Egyptian Journal of Forensic Sciences, Volume 5, Issue 4, 2015, 157-161

RESPONSE TO REVIEWERS

Dear Academic Editor

Prof. Kapil Amgain

thank you very much for your kind mail and the precious reviews included in your response.

We are glad to inform You that all the criticisms raised by Reviewers have been accepted.

The manuscript has been largely revised and improved according to these criticisms.

In this regard, we really thank the Reviewers for the relevant improvement to the article

This is our reply point-by-point.

Reviewer #1

a) Reply to issue no. 1: "*Dear author the article you have cited here is speaking of only orbital index and that too not about evoution. please revise and be specific while citing articles*"

   Thank you for your remark. We have added the following references:

   - Oladipo GS, Olabiyi AO, Oremosu AA, NoronhaCC. Nasal indices among major ethnic groups in Southern Nigeria. SRE. 2007; 2(1): 20-22.
   - Ravichandran S, Yuvraj Babu K, Mohanraj KG. Correlation of facial and nasal index in gender determination. Drug Invention Today. 2018; 10(12).

b) Reply to issue no. 2: "*What were the inclusion and exclusion criteria of the study?*"

   In the "Materials and Methods" section, we have added the following explanation: "Inclusion criteria were Kosovar males and females over the age of 18, who underwent CT scan to investigate neurological symptoms. Exclusion criteria included growth diseases, endocrine disorders or osteodystrophy, previous fractures of the of the skull and the facial massif. Finally, all blurred TC images were disregarded"

c) Reply to issue no. 3: "*How was this sample size calculated?*"

   In the "Materials and Methods" section, we have added the following explanation: "The sample of the present study was calculated based on the effects observed in the study by Kotian et al. From this study, the mean effect size of difference between groups of 0.20 (Cohen's d) was observed. Adopting a 5% type I error (95% study confidence level), a 20% type II error (80% study power), the effect size observed in the mentioned study and, considering the comparison tests of the groups of the bilateral type (two-sided), it was calculated that at least 394 sample units were necessary for the accomplishment of the study. As they are available, we conducted the study with 408 sample units. The calculations were performed using the G*Power statistical software"

d) Reply to issue no. 4: "*Dear author, is this line so important for the result section.*"

Thanks for the suggestion. We have deleted the previous sentence and explained our thinking better: "It was not possible to fit a multiple model, with more than one variable being a predictor together with another, among the variables evaluated. For this reason, the univariate analysis is shown in Table 2, where it is observed that, in isolation, the variables NW and NH were configured as predictors of sex"

e) Reply to issue no. 5: "This is not a correct statement over here, the increase in the number of samples depends upon the sample size calculation and in this study the authors have enough sample size. however, the basis of sample taken has not been shown so the methodology clearly needs how the sample size was obtained and then the revision of the line 263 has to be thought of"

The following sentence has been deleted "to increase the number of samples". The answer to criticism No. 3 shows how the sample was calculated.

f) Reply to issues nos. 6, 7, 8, 9 and 10.

The references have been corrected

***

Reviewer #2

a) Reply to issue no. 1: "*Can you make clear why you choose female sex as a reference group?*"

We have added the following sentence to better explain the reason for the choice: "The female gender was chosen as the reference category because the measures of interest were lower for females and leaving this category as a reference makes the interpretation of the odds ratio findings more understandable"

b) Reply to issues nos. 2 and 3: "*The authors did not establish a correlation between sex difference and mean age why?*" and "*The authors did not establish a correlation between sex difference and mean age why?*"

Thanks to the reviewer for the remark. The comparison was made and reported in the 'Results' section. Below is the sentence: "251 individuals were evaluated, 110 females and 141 males, with a mean age of 37.41 (±20.56) and 33.87 (±19.58), respectively, there was no statistically significant difference in age between the sexes. (p=0.232; Student's t-test)".

c) Reply to issue no. 4: "*Besides forensic identification, please add some other reasons why facial measurements are necessary*"

In the "Discussion" section, we have added the following sentence (with reference) to better explain some other reasons why facial measurements are necessary: "Face measurements are also made for various health purposes, such as in the field of dental prostheses, cosmetic surgery,

orthodontics and face masks. In particular, face analysis and proportions have been shown to be important for facial plastic surgeons to evaluate the face during the planning stages of facial reconstructive and cosmetic surgery

- Shahbazi Z, Ardalani H, Maleki M. Aesthetics of Numerical Proportions in Human Cosmetic Surgery. World J Plast Surg. 2019 Jan;8(1):78-84.

d) Reply to issue no. 5: "*Why not to compare the finding of CT scan with direct measurement of some subjects to verify whether the measurement is affected by the magnification factor or not?*"

   Good observation, this study is in anticipation. In the conclusions section we added this sentence: "In the future, studies could be conducted to compare the measurements made on CT scans with those performed directly on the skulls and verify if there is an anthropometric variability that could make the results different"

For details we invite You to check the file revised and updated in attachment.

We really hope that the comments raised by the Reviewers have been satisfied in this manner.

We look forward to hearing from You.

Sincerely,

Dr. Francesco De Micco, M.D., Ph.D

Corresponding author
