# Supplementary material for: Anthropometric analysis of orbital and nasal parameters for sexual dimorphism: New anatomical evidences in the field of personal identification through a retrospective observational study
Source: PLoS One. 2023 May 3;18(5):e0284219. doi: 10.1371/journal.pone.0284219 (PMC10155994; doi:10.1371/journal.pone.0284219)
Supplement: Attachment — Submitted filename: Response to Reviewers.docx [file pone.0284219.s004.docx]

**RESPONSE TO REVIEWERS**

*Dear Academic Editor*

*Prof. Kapil Amgain*

thank you very much for your kind mail and the precious reviews included in your response.

We are glad to inform You that all the criticisms raised by Reviewers have been accepted.

The manuscript has been largely revised and improved according to these criticisms.

In this regard, we really thank the Reviewers for the relevant improvement to the article

This is our reply point-by-point.

**Reviewer #1**

a) Reply to issue no. 1: “*Dear author the article you have cited here is speaking of only orbital index and that too not about evoution. please revise and be specific while citing articles*”

Thank you for your remark. We have added the following references:

- Oladipo GS, Olabiyi AO, Oremosu AA, NoronhaCC. Nasal indices among major ethnic groups in Southern Nigeria. SRE. 2007; 2(1): 20-22.
- Ravichandran S, Yuvraj Babu K, Mohanraj KG. Correlation of facial and nasal index in gender determination. Drug Invention Today. 2018; 10(12).

b) Reply to issue no. 2: “*What were the inclusion and exclusion criteria of the study?*”

In the “Materials and Methods” section, we have added the following explanation: “Inclusion criteria were Kosovar males and females over the age of 18, who underwent CT scan to investigate neurological symptoms. Exclusion criteria included growth diseases, endocrine disorders or osteodystrophy, previous fractures of the of the skull and the facial massif. Finally, all blurred TC images were disregarded”

c) Reply to issue no. 3: “*How was this sample size calculated?*”

In the “Materials and Methods” section, we have added the following explanation: “The sample of the present study was calculated based on the effects observed in the study by Kotian et al. From this study, the mean effect size of difference between groups of 0.20 (Cohen's d) was observed. Adopting a 5% type I error (95% study confidence level), a 20% type II error (80% study power), the effect size observed in the mentioned study and, considering the comparison tests of the groups of the bilateral type (two-sided), it was calculated that at least 394 sample units were necessary for the accomplishment of the study. As they are available, we conducted the study with 408 sample units. The calculations were performed using the G*Power statistical software”

d) Reply to issue no. 4: “*Dear author, is this line so important for the result section.*”

Thanks for the suggestion. We have deleted the previous sentence and explained our thinking better: “It was not possible to fit a multiple model, with more than one variable being a predictor together with another, among the variables evaluated. For this reason, the univariate analysis is shown in Table 2, where it is observed that, in isolation, the variables NW and NH were configured as predictors of sex”

e) Reply to issue no. 5: “This is not a correct statement over here, the increase in the number of samples depends upon the sample size calculation and in this study the authors have enough sample size. however, the basis of sample taken has not been shown so the methodology clearly needs how the sample size was obtained and then the revision of the line 263 has to be thought of”

The following sentence has been deleted “to increase the number of samples”. The answer to criticism No. 3 shows how the sample was calculated.

f) Reply to issues nos. 6, 7, 8, 9 and 10.

The references have been corrected

***

**Reviewer #2**

a) Reply to issue no. 1: “*Can you make clear why you choose female sex as a reference group?*”

We have added the following sentence to better explain the reason for the choice: “The female gender was chosen as the reference category because the measures of interest were lower for females and leaving this category as a reference makes the interpretation of the odds ratio findings more understandable”

b) Reply to issues nos. 2 and 3: “*The authors did not establish a correlation between sex difference and mean age why?*” and “*The authors did not establish a correlation between sex difference and mean age why?*”

Thanks to the reviewer for the remark. The comparison was made and reported in the 'Results' section. Below is the sentence: “251 individuals were evaluated, 110 females and 141 males, with a mean age of 37.41 (±20.56) and 33.87 (±19.58), respectively, there was no statistically significant difference in age between the sexes. (p=0.232; Student’s t-test)”.

c) Reply to issue no. 4: “*Besides forensic identification, please add some other reasons why facial measurements are necessary*”

In the "Discussion" section, we have added the following sentence (with reference) to better explain some other reasons why facial measurements are necessary: “Face measurements are also made for various health purposes, such as in the field of dental prostheses, cosmetic surgery, orthodontics and face masks. In particular, face analysis and proportions have been shown to be important for facial plastic surgeons to evaluate the face during the planning stages of facial reconstructive and cosmetic surgery

- Shahbazi Z, Ardalani H, Maleki M. Aesthetics of Numerical Proportions in Human Cosmetic Surgery. World J Plast Surg. 2019 Jan;8(1):78-84.

d) Reply to issue no. 5: “*Why not to compare the finding of CT scan with direct measurement of some subjects to verify whether the measurement is affected by the magnification factor or not?*”

Good observation, this study is in anticipation. In the conclusions section we added this sentence: “In the future, studies could be conducted to compare the measurements made on CT scans with those performed directly on the skulls and verify if there is an anthropometric variability that could make the results different”

For details we invite You to check the file revised and updated in attachment.

We really hope that the comments raised by the Reviewers have been satisfied in this manner.

We look forward to hearing from You.

Sincerely,

Dr. Francesco De Micco, M.D., Ph.D

Corresponding author
