# Supplementary material for: Anthropometric analysis of orbital and nasal parameters for sexual dimorphism: New anatomical evidences in the field of personal identification through a retrospective observational study
Source: PLoS One. 2023 May 3;18(5):e0284219. doi: 10.1371/journal.pone.0284219 (PMC10155994; doi:10.1371/journal.pone.0284219)
Supplement: Attachment — Submitted filename: Comments-PONE-D-22-09865 (2).docx [file pone.0284219.s007.docx]

**Reviewer comments:**

1. **General response to the research:**
a. This study aimed to ascertain whether there are sex differences in the orbital and/or nasal indexes and/or the single measurements used to calculate these variables in a Kosovar population. Craniometric parameters including NI and OI have been investigated in previous studies in forensic medicine. However, the literature shows that there are significant differences in these parameters between individuals in relation to age, sex, and ethnicity. Although, this study does not show any novel techniques and methods, it did study a specific population (Different ethnicity) which is the Kosovar population.
b. The methodology in this study is clear with some suggestions to be addressed by the authors. The overall Results and discussion are good. English language used is very good. There are some problems with the references in the references section.
c. The supplementary data or findings were not provided. No link or deposition to a public repository was found in the submitted manuscript, although the authors mentioned “Yes - all data are fully available without restriction”
d. **It is better to follow the STROBE guidelines in reporting data for retrospective studies.**
e. **This article, in my opinion, suits this journal’s aim and vision.**
f. **All the comments were highlighted in the pdf starting from page 44.**
2. **Title:** Mention the type of study at the end of the title.
3. **Abstract:**

a. Please re-write the abstract according to the journal guidelines.

b. Kindly delete the abbreviations that are not useful.

c. Please write CT in full.

4. **Introduction:**
a. No hypothesis was presented in this section.
b. Please add reference for the statement that end in row 65.
c. Please cite the studies that you have mentioned (The sentence in row 66 and 67)
5. **Methodology:** The authors did not follow completely STROBE guidelines in reporting their methodology.
   a. Describe the setting, locations, and relevant dates of selecting and measuring the data.
   b. State the Ethical approval in the beginning of this section. This study involving medical records. so Ethical approval by a review board is needed.
   c. Please add the ethnic group in your inclusion criteria.
   d. Correct the mistake in row 86
   e. In row 93, please size to the sample.
   f. State any risk of potential bias if necessary (the difference in the sample size of the initial test phase between males (n=141) and females (n=110)).
   g. How did you manage any possible risk of the X-ray machine magnification? Was there any criteria when selecting a proper x-ray to be studied?
   h. In figure 1, please state the dimensions of the film and indicate which side is the left or right.
6. **Results:**
   a. Please add this in the sentence of row 146. " 251 individuals were evaluated at the initial test phase of the study"
   b. In row 164, please change predictive to likelihood
7. **Discussion:** Please add a discussion on your hypothesis depending on what you did write in the introduction section.
8. **Conclusion:** Please remove the 40-42 or add them into the discussion. It is advisable not to include any new references here as the conclusion is withdrawn from your data and discussion.
9. **References:** Few mistakes were found such as page number, journal name, etc. Kindly check the journal guidelines again before you do any corrections. Some mistakes were highlighted in the pdf. Kindly check all the references and correct whenever needed.
10. **Figures:** In figure 1, please state the dimensions of the film and indicate which side is the left or right.
