# Supplementary material for: Anthropometric analysis of orbital and nasal parameters for sexual dimorphism: New anatomical evidences in the field of personal identification through a retrospective observational study
Source: PLoS One. 2023 May 3;18(5):e0284219. doi: 10.1371/journal.pone.0284219 (PMC10155994; doi:10.1371/journal.pone.0284219)
Supplement: Attachment — Submitted filename: Response to Reviewers.docx [file pone.0284219.s009.docx]

**RESPONSE TO REVIEWERS**

*Dear Academic Editor*

thank you very much for your kind mail and the precious reviews included in your response.

We are glad to inform You that all the criticisms raised by Reviewers have been accepted.

The manuscript has been revised and improved according to these criticisms.

In this regard, we really thank the Reviewers for the relevant improvement to the article

This is our reply point-by-point.

**Reviewer #1**

Dear Reviewer #1,

all authors are grateful to you for considering our manuscript interesting and for your suggestions.

We accepted your proposals and implemented our manuscript.

a) **Reply to issue: “*On line no 146, please do not start your result section with numeral (251) rather it should be Two hundred fifty one*”.**

Thank you for your remark. Taking up the suggestion, we have changed from numbers to letters

**Reviewer #3**

Dear Reviewer #3,

all authors are grateful to you for considering our manuscript interesting and for your suggestions.

We accepted your proposals and implemented our manuscript.

a) **Reply to issue no. 1c: “*The supplementary data or findings were not provided. No link or deposition to a public repository was found in the submitted manuscript, although the authors mentioned “Yes - all data are fully available without restriction*”**

We have uploaded an excel file as supplementary data

b) **Reply to issue no. 1d: “*It is better to follow the STROBE guidelines in reporting data for retrospective studies*”.**

We have modified according to STROBE guidelines

c) **Reply to issue no. 2: “*Please mention the type of study at the end of the title*”.**

We have added “through a retrospective observational study” in the title

d) **Reply to issue no. 3: “*Abstract: a. Please re-write the abstract according to the journal guidelines. b. Kindly delete the abbreviations that are not useful. c. Please write CT in full*”**

The abstract was rewritten with the aim of (i) describe the main objective of the study; (i) explain how the study was done, including any model organisms used, without methodological detail; (i) summarize the most important results and their significance; (i) not exceed 300 words. All this according to Plosone guidelines.

e) **Reply to issue no. 4a: “*No hypothesis was presented in this section*”**

We have stated our research hypothesis by adding the following sentence: “The authors assume that there may be differences between the two sexes at least in one or more parameters necessary to calculate either the nasal index or the orthotic index in the population under examination. If the hypothesis is confirmed, on the basis of the results obtained, the authors will provide a plausible explanation in this regard”.

f) **Reply to issue no. 4b: “*Please add reference for the statement that end in row 65.*”**

The following reference is made to the sentence: Cameron N, Lones LL. Growth, maturation and age, in: Black S, Aggrawal A, Payne-James J, editors. Age estimation in the living. The practitioners guide. Chichester, West Sussex, UK; 2010. pp. 95-120.

g) **Reply to issue no. 4c: “*Please cite the studies that you have mentioned (The sentence in row 66 and 67)*”**

The following references are made to the sentence:

- Botwe BO, Sule DS, Ismael AM. Radiologic evaluation of orbital index among Ghanaians using CT scan. J Physiol Anthropol. 2017;36:29.
- Gupta V, Prabhakar A, Yadav M, Khandelwal N. Computed tomography imaging-based normative orbital measurement in Indian population. Indian J Ophthalmol. 2019; 67:659–63.
- Iscan M. Forensic anthropology of sex and body size. Forensic Sci Int. 2005; 147:107–12
- Russel SM, Frank-Ito DO. Gender Differences in Nasal Anatomy and Function Among Caucasians. Facial Plast Surg Aesthet Med. 2023; 2:145-152

h) **Reply to issue no. 5a: “*Describe the setting, locations, and relevant dates of selecting and measuring the data*”.**

We reworked the sentence: “The CT scans were anonymously extracted and limited data were extracted that were strictly necessary and relevant for the conduct of the study. The extracted data are all available in the manuscript. CT examinations were analyzed from 1 February to 15 March 2022”

i) **Reply to issue no. 5b: “*State the Ethical approval in the beginning of this section. This study involving medical records. so Ethical approval by a review board is needed*”**

We reworked the sentence: “The study was approved by the Ethical Issues Committee of the Kosovo Chamber of Dentists (approval no. 27 of 18 May 2022)”.

The Ethics Committee's approval document was submitted

j) **Reply to issue no. 5c: “*Please add the ethnic group in your inclusion criteria*”**

We reworked the sentence: “The inclusion criteria were males and females of Kosovar ethnicity …”

k) **Reply to issue no. 5d: “*Correct the mistake in row 86*”**

TC has been corrected in CT

l) **Reply to issue no. 5e: “*In row 93, please size to the sample*”**

Addition done

m) **Reply to issue no. 5f: “*State any risk of potential bias if necessary (the difference in the sample size of the initial test phase between males (n=141) and females (n=110))*”**

We added the sentence: “The random splitting of the sub-samples could represent a potential research bias”

n) **Reply to issue no. 6f: “*How did you manage any possible risk of the X-ray machine magnification? Was there any criteria when selecting a proper x-ray to be studied?*”**

Regarding the possibility of magnification, the CTs were performed in standard projections. Therefore, we have a reasonable certainty that no interpretative alterations are possible. In addition, the software used guarantees perfect measurement in millimetres.

This certainty is also the result of the exclusion criteria. And here, we answer the second question: all CTs of patients with growth disorders, endocrine disorders or osteodystrophy, previous fractures of the skull and facial massif were excluded.

o) **Reply to issue no. 5h: “*In figure 1, please state the dimensions of the film and indicate which side is the left or right*”.**

With regard to size, only measurements of interest were taken for the parameters required for the study (orbital and nasal). As for the right and left side, these are common CT images in antero-posterior projection.

p) **Reply to issue no. 6a: “*Please add this in the sentence of row 146. "251 individuals were evaluated at the initial test phase of the study*".**

We reworked the sentence: “Two hundred fifty-one individuals were evaluated at the initial test phase of the study”

q) **Reply to issue no. 6b: “*In row 164, please change predictive to likelihood*”**

We reworked the sentence: “Table 3 also shows the positive and negative likelihood values (LR+ and LR-) …”

r) **Reply to issue no. 7: “*Discussion: Please add a discussion on your hypothesis depending on what you did write in the introduction section*”**

We have added the following sentence: “In confirmation of the hypothesis that initiated this research, the statistically significant differences found between the two sexes as regards the nasal parameters could be traced back to a different process of bone growth of parts of the facial mass in males and females, due both to genetic factors typical of a specific population group and/or evolutionary-environmental factors established over time”.

s) **Reply to issue no. 8: “*Please remove the 40-42 or add them into the discussion. It is advisable not to include any new references here as the conclusion is withdrawn from your data and discussion*”.**

Indeed, these references are more suitable for introduction.

t) **Reply to issue no. 9: “*Few mistakes were found such as page number, journal name, etc. Kindly check the journal guidelines again before you do any corrections. Some mistakes were highlighted in the pdf. Kindly check all the references and correct whenever needed*”.**

We revised the layout of all references

u) **Reply to issue no. 10: “*Figures: In figure 1, please state the dimensions of the film and indicate which side is the left or right”***

Please kindly compare with the answer to point “n”,

**Reviewer #4**

Dear Reviewer #4,

all authors are grateful to you for considering our manuscript interesting and for your suggestions.

We accepted your proposals and implemented our manuscript.

a) **Reply to issue no. 1: “*Reviewer #2 has asked regarding age and a response has been given though the range is quite big. Is it possible for the authors to do analysis on a 5 year or 10 year age range of individuals disregarding the sex? Example 18-27 years old, 28-37 ..... There should be a difference in the bone morphology of elderly adults compared to 18 year old healthy individuals. Based on the raw data obtained, there should be enough to just*”.**

Thank you for your remark. The observation is very pertinent. However, as it stands, the study has the limitations mentioned at the end of the paper. Specifically, the study showed moderate accuracy with not very high sex classification power. We should understand in the future whether this nasal anatomical variation is due to the adaptation of the Kosovar population to the specific environment or whether it affects other ethnic groups, in general.

Therefore, we also enthusiastically welcome and will evaluate in future studies the aspect suggested by reviewer #4 to see if and how bone variability might impact the method.

For this reason, we have added the following sentence: “Furthermore, for the purpose of forensic identification, it will be useful to verify any anthropometric differences in relation to age, as well as to supplement the method with other already known or innovative methods for age estimation”

b) **Reply to issue no. 2: “*The paper mentions exclusion of pathological deformed bones. Does edentulous fall under this category? The maxilla bone can appear normal, but the person may not have maxilla teeth. Please refer to this paper for why the orbit is affected by aging: Pessa, J.E., 2000. An algorithm of facial aging: verification of Lambros’s theory by three-dimensional stereolithography, with reference to the pathogenesis of midfacial aging, scleral show, and the lateral suborbital trough deformity. Plastic and reconstructive surgery, 106(2), pp.479-488*”**

We had no cases of edentualia. However, they would not have been excluded. The exclusion criteria were made explicit in the study and concern: growth diseases, endocrine disorders or osteodystrophy, previous fractures of the of the skull and the facial massif.

We gladly read the suggested paper (Pessa JE, 2000) and included it to make our discussion on age-related morphological variability even more 'evidence-based'.

For details we invite You to check the file revised and updated in attachment.

We really hope that the comments raised by the Reviewers have been satisfied in this manner.

We look forward to hearing from You.

Sincerely,

Dr. Francesco De Micco, M.D., Ph.D

Corresponding author
